# Global research landscape and trends of lung cancer immunotherapy: A bibliometric analysis

**DOI:** 10.3389/fimmu.2022.1032747

**Published:** 2022-12-01

**Authors:** Yanhao Liu, Xu Cheng, Xiaona Han, Xi Cheng, Shu Jiang, Yaru Lin, Zhen Zhang, Linlin Lu, Baozhen Qu, Yuxian Chen, Xiaotao Zhang

**Affiliations:** ^1^ Department of Radiation Oncology, The Affiliated Qingdao Central Hospital of Qingdao University, Qingdao, China; ^2^ School of Basic Medicine, Qingdao University, Qingdao, China

**Keywords:** bibliometric analysis, lung cancer, PD1/PDL1, clinical trials, pembrolizumab, nivolumab, immunotherapy, tumor mutation burden

## Abstract

**​Background:**

Immunotherapy for lung cancer has been a hot research area for years. This bibliometric analysis aims to present the research trends on lung cancer immunotherapy.

**Method:**

On 1 July, 2022, the authors identified 2,941 papers on lung cancer immunotherapy by the Web of Science and extracted their general information and the total number of citations. A bibliometric analysis was carried out to present the research landscape, demonstrate the research trends, and determine the most cited papers (top papers) as well as major journals on lung cancer immunotherapy. After that, recent research hotspots were analyzed based on the latest publications in major journals.

**Results:**

These 2,941 papers were cited a total of 122,467 times. “Nivolumab vs. docetaxel in advanced non–squamous non–small–cell lung cancer” published in 2015 by Borghaei H et al. was the most cited paper (5,854 citations). Among the journals, *New England Journal of Medicine* was most influential. Corresponding authors represented China took part in most articles (904) and papers with corresponding authors from the USA were most cited (139.46 citations per paper). Since 2015, anti–PD–(L)1 has become the hottest research area.

**Conclusions:**

This bibliometric analysis comprehensively and quantitatively presents the research trends and hotspots based on thousands of publications, and further suggests future research directions. Moreover, the results can benefit researchers to select journals and find potential collaborators. This study can help researchers get a comprehensive impression of the research landscape, historical development, and recent hotspots in lung cancer immunotherapy and provide inspiration for further research.

## Introduction

In recent decades, lung cancer has always become one of the most commonly diagnosed cancers and the leading cause of cancer–related deaths worldwide (1). As a heterogeneous disease, lung cancer is classified as non–small cell lung cancer (NSCLC, ~85%) and small cell lung cancer (SCLC, ~15%) (1). The main treatments for lung cancer involved surgery, radiotherapy, and chemotherapy. In recent years, immune checkpoint inhibitors (ICIs) and targeted therapy have led to a remarkable improvement in the prognosis of patients with lung cancer (2). Programmed cell death 1 (PD–1)/PD1 ligand 1 (PD–L1) interaction is the most frequent target for lung cancer immunotherapy. Blocking this interaction by ICIs leads to increased T–cell activation and enhanced anti–tumor immunity (3). Currently, anti–PD–(L)1 antibodies monotherapy or combined with other therapies have become standard treatments for a large portion of patients with lung cancer, especially patients with advanced lung cancer (1). Lung cancer immunotherapy has been a rapidly growing research area since 2010, with hundreds of articles published every year. It is necessary but challenging for researchers to master research trends and monitor the latest important advances. Therefore, a comprehensive and quantified analysis is required that systemically summarizes important advances, presents the latest research hotspots, and suggests research directions.

Bibliometric analysis is suitable for the comprehensive evaluation of an entire academic discipline including thousands of publications, whereas other major review methods are not (4). Based on the quantitative analysis of structured information from relevant publications, a bibliometric analysis can objectively describe the landscape, research trends, and research hotspots (5). The results are helpful to the researchers in defining the progress of the filed, determining research direction, identifying collaborators, and selecting a target journal for publication (6). ​Thus, bibliometrics is well suited to quantitatively analyze research trends in lung cancer immunotherapy. In recent years, bibliometric analysis has become increasingly popular in medical research (7, 8). Two bibliometric analyses related to lung cancer immunotherapy have been published (2, 9). However, the first included only papers published before March 2020, which is more than 2 years before the present study was conducted (9). The latter analyzed all articles related to anti–PD–(L)1 for cancer immunotherapy, without limitation of the type of cancer (2). Furthermore, both studies only analyzed the 100 most cited articles, and articles focused on immunotherapy outside of anti–PD–(L)1 were not included. Therefore, a comprehensive, up–to–date, and useful bibliometric analysis of lung cancer immunotherapy is necessary.

The present bibliometric analysis analyzed original articles directly related to clinical immunotherapy for lung cancer published from 2010 to 1 July 2022 and identified the 100 most frequently cited articles (top papers). Furthermore, an additional bibliometric analysis was conducted based on the latest major publications to indicate the latest research hotspots. The objective of this study was to present a comprehensive landscape, research trends, important advances, and current hotspots for researchers. Based on this study, researchers can not only identify key publications, journals, and potential collaborators but may also be stimulated to design more studies.

## Methods

### Database and paper selection

The Web of Science Core Collection (WoSCC) is one of the most frequently used databases for bibliometric analysis, including more than 10,000 high–quality journals and comprehensive citation records (2). Moreover, the WoSCC document type labels have been shown to be more precise than other databases such as Scopus (10). In this study, we selected the WoSCC Science Citation Index Expanded database for the literature search.

**Figure 1:**
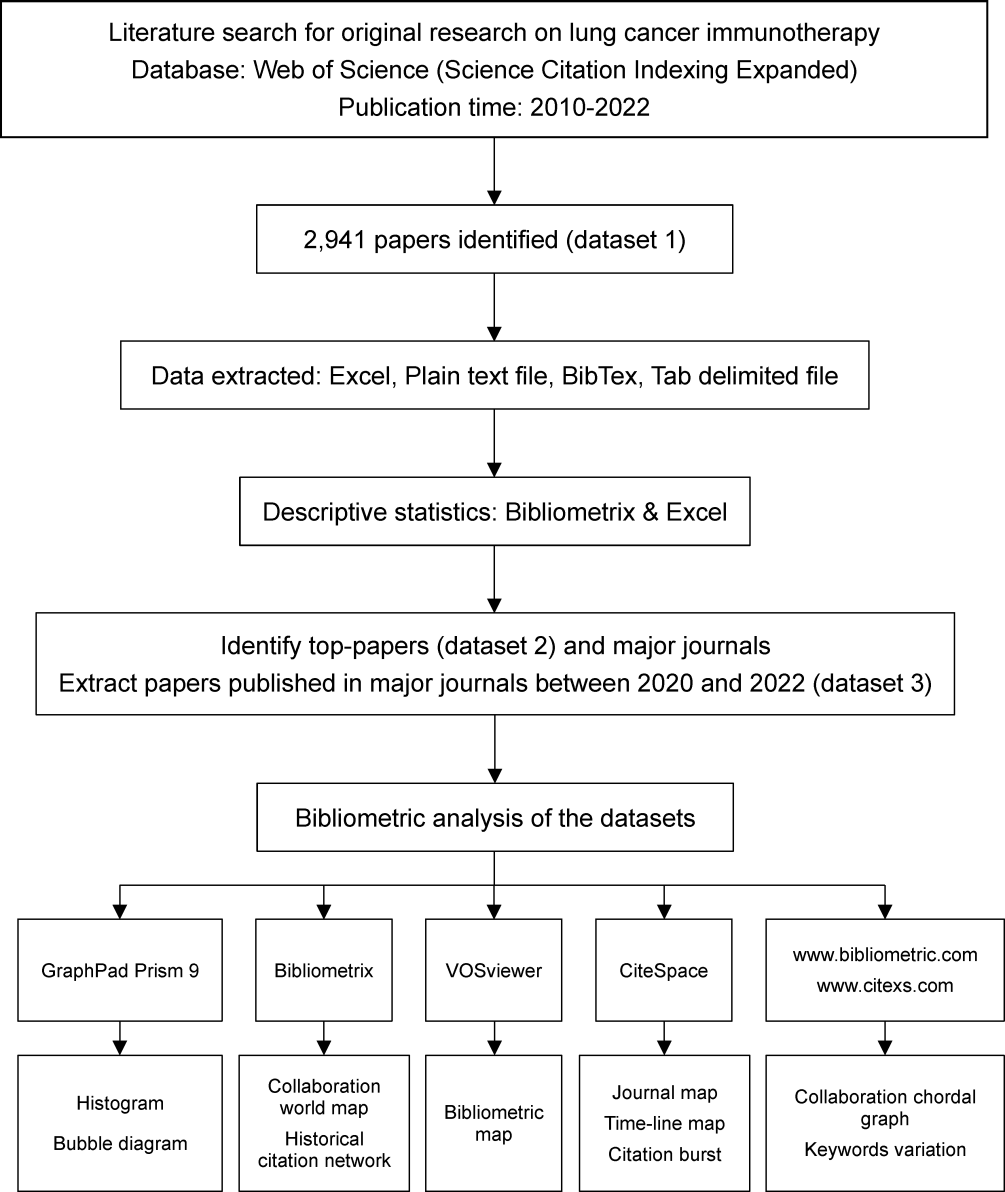
The workflow of the present study.

The workflow of this study was shown in (**Figure 1**). A literature search was conducted on 1 July 2022 for original research articles on lung cancer immunotherapy published since 2010. The authors designed the search strategy as follows to include as many relevant papers as possible while excluding irrelevant papers: 1) keywords were searched only in titles, because some irrelevant papers may contain the search keywords in abstracts; 2) the keywords were “lung cancer” and “immunotherapy”; 3) synonyms of keywords were included as much as possible, and synonyms of “immunotherapy” included specific names of drugs or treatments; 4) papers outside of original studies were excluded; 5) papers containing the term ‘lung metastasis’ were excluded, because these papers were not related to primary lung cancer. The authors performed multiple tests and modifications to ensure the sensitivity and specificity of the search strategy. The detailed search strategy is presented in **Supplementary Material S1**.

The relevant articles were then identified, and the following information was extracted: title; abstract; keywords; authors; publication time; journal; countries/regions; institutions; the total number of citations; and average number of citations per year (calculated as the number of citations per month × 12). The authors then ranked the papers with the number of citations to identify the 100 top papers.

### Statistical analysis

Microsoft Office Excel 2019 software (Microsoft, Redmond, WA, USA) was used for descriptive statistical analysis, correlation test, and to produce tables. The GraphPad Prism 9 software (Dotmatics, San Diego, CA, USA) was used for plot histograms and bubble diagrams. The “bibliometrix” package was an open–source tool for performing comprehensive science mapping analysis (11). Bibliometrix of R software (v4.1.2) was used for bibliometric analysis and data visualization. VOSviewer (Leiden University, Netherlands) was a software for constructing and viewing bibliometric maps and could display large bibliometric maps in an easy–to–interpret way (12). VOSviewer (v1.6.17) was used to construct bibliographic coupling networks of journals, countries, coauthors, and keywords. Using a customized VOSviewer thesaurus file, the authors merged the synonyms and different derivatives of keywords, countries, and coauthors to better present the networks. The words in VOSviewer networks defaulted to lowercase letters, and the author capitalized some letters to standardize writing. An online platform (https://bibliometric.com) was used to visualize cooperation between countries/regions, and another online platform (https://www.citexs.com) was used to visualize the trends of keyword frequencies. CiteSpace software (v6.1.R1) was used to detect keywords and references with the strongest citation bursts, to construct visualization maps of co–cited references and keywords, and to plot a dual–map overlay of journals. To indicate and visualize the research trends in lung cancer immunotherapy from 2010 to 2022, the authors classified the articles by searching for specific therapies and treatment lines in titles and abstracts. Microsoft Visual Basic for Applications was used to perform a macro for data arrangement and batch retrieval.

To determine whether papers published in highly indexed journals were more cited, the authors conducted a correlation test between average citation per paper per year and (1) 5–year impact factor (IF) and (2) Journal Citation Index (JCI) of journals with a 5–year IF > 5 published at least two papers on lung cancer immunotherapy.

The journals that published the top papers were identified, and their top papers rates (TPR, the percentage of top papers among all relevant papers in a journal) were calculated. Journals with a TPR >5% were considered the top journals on lung cancer immunotherapy. The papers on lung cancer immunotherapy published in top journals since 2020 were identified and analyzed to evaluate recent research hotspots.

## Results

**Figure 2:**
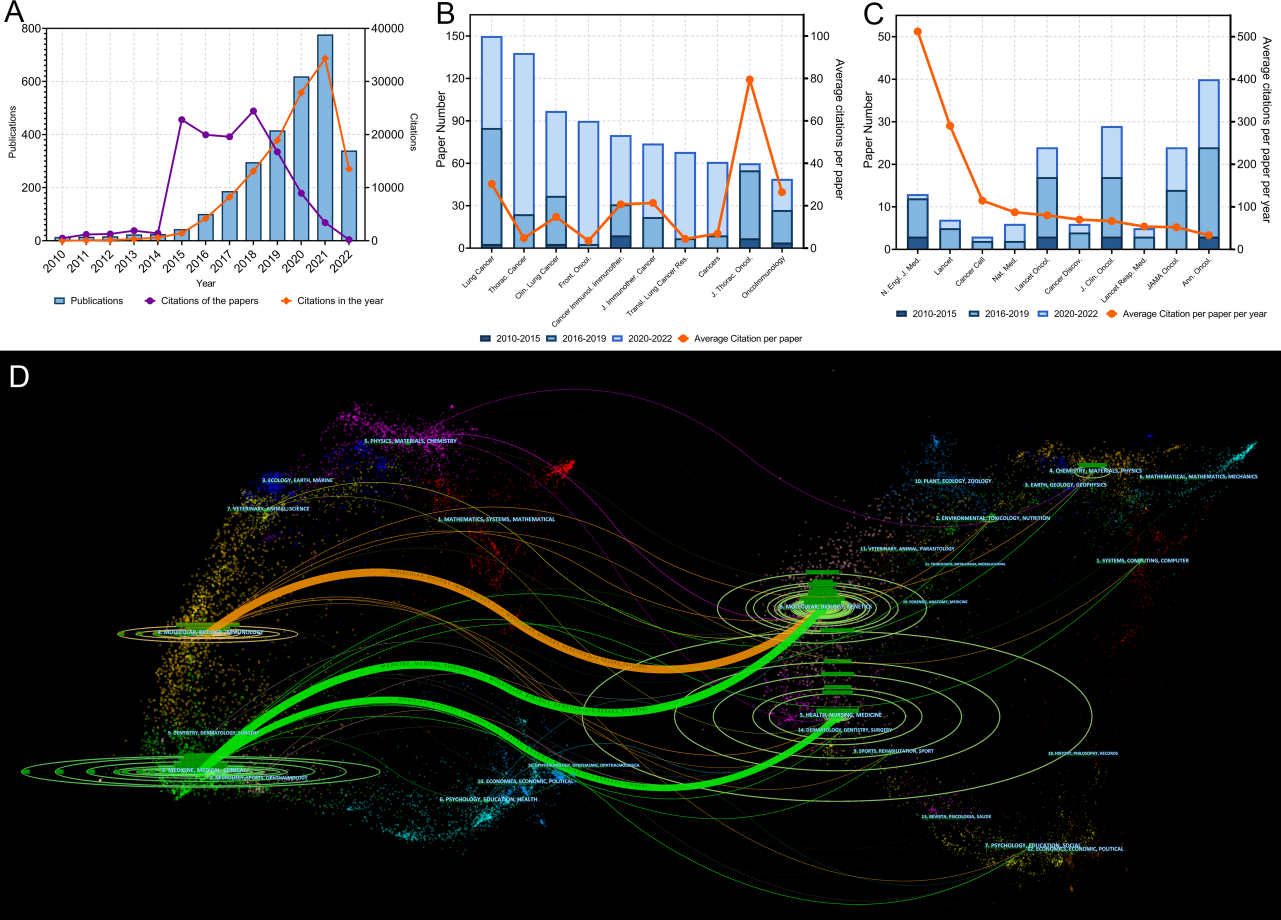
**(A)** Publication and citation number from 2010 to 2022 of the papers on lung cancer immunotherapy. The purple line indicates the total citations of papers published each year. The orange line indicates the total citations of all papers each year. **(B)** Paper numbers and average citations per paper of the top-10 productive journals. **(C)** Top-10 journals with the most citations per paper per year. **(D)** The dual-map overlay of journal categories. The left nodes represent citing journals and the right nodes represent cited journals. The curves represent the citation relationship.

The literature search yielded 2,941 original articles on lung cancer immunotherapy published between 2010 and 1 July 2022 (**Figure 2A**). In recent years, the number of articles published each year has grown rapidly. More than 90% of the articles were published after 2015, while more than 50% of the articles were published after 2019. These articles were cited 122,467 times and the median number of citations was 7. Although only 44 papers were published in 2015, they were cited 22,806 times. To present the citation relationship among the key–node papers, a historical direct–citation network was plotted (**Supplementary Figure S1**). The 25 main references with the strongest citation bursts are listed in **Supplementary Figure S2**. The bibliographic coupling network of the most co–cited references is shown in **Supplementary Figure S3**.

The authors ranked the articles with the citation number and identified 100 top papers (**Supplementary Table S1**). The authors also identified the 100 papers with highest citation per year (**Supplementary Table S2**). The top papers were cited 76,556 times, which was 62.5% of the number of articles cited on lung cancer immunotherapy. The median number of citations in the top papers was 383.5 (range: 165–5,854). “Nivolumab versus docetaxel in advanced non–squamous non–small–cell lung cancer” published in 2015 by Borghaei et al. had the highest number of citations (5,854) and the second highest average number of citations per year (878.1) (13). “Pembrolizumab versus chemotherapy for PD–L1–positive non–small–cell lung cancer” published in 2016 by Reck et al. had the second highest number of citations (5,287) and the highest average number of citations per year (926.9) (14). Among the 10 most cited articles, 7 were published in the *New England Journal of Medicine* (*N Engl J Med*) (**Table 1**). Most of the top papers (66 papers) were published between 2016 and 2018. Only 3 top papers were published in 2020 or 2021. “First–line nivolumab plus ipilimumab combined with two cycles of chemotherapy in patients with non–small–cell lung cancer (CheckMate 9LA): an international, randomised, open–label, phase 3 trial” published in 2021 by Paz–Ares et al. was the latest top paper, which had a citation number of 215 and an average citation per year of 161.3 (15).

**Table 1:** The 10 most cited papers in lung cancer immunotherapy from 2010 to 2022^a^.

Rank	Title	CorrespondingAuthor	Journal	Year	Total citations	Average citations per year (rank)
1	Nivolumab versus Docetaxel in Advanced Nonsquamous Non-Small-Cell Lung Cancer	Borghaei H	N Engl J Med	2015	5854	878.1 (2)
2	Pembrolizumab versus Chemotherapy for PD-L1-Positive Non-Small-Cell Lung Cancer	Brahmer JR	N Engl J Med	2016	5287	946.9 (1)
3	Mutational landscape determines sensitivity to PD-1 blockade in non-small cell lung cancer	Chan TA	Science	2015	4908	684.8 (4)
4	Nivolumab versus Docetaxel in Advanced Squamous-Cell Non-Small-Cell Lung Cancer	Brahmer J	N Engl J Med	2015	4612	666.8 (5)
5	Pembrolizumab for the Treatment of Non-Small-Cell Lung Cancer	Garon EB	N Engl J Med	2015	3833	541.1 (6)
6	Pembrolizumab versus docetaxel for previously treated, PD-L1-positive, advanced non-small-cell lung cancer (KEYNOTE-010): a randomised controlled trial	Herbst RS	Lancet	2016	3217	521.7 (7)
7	Pembrolizumab plus Chemotherapy in Metastatic Non-Small-Cell Lung Cancer	Gandhi L	N Engl J Med	2018	2827	692.3 (3)
8	Atezolizumab versus docetaxel in patients with previously treated non-small-cell lung cancer (OAK): a phase 3, open-label, multicentre randomised controlled trial	Gandara DR	Lancet	2017	2647	488.7 (8)
9	Durvalumab after Chemoradiotherapy in Stage III Non-Small-Cell Lung Cancer	Antonia SJ	N Engl J Med	2017	2002	436.8 (10)
10	Pembrolizumab plus Chemotherapy for Squamous Non-Small-Cell Lung Cancer	Paz-Ares L	N Engl J Med	2018	1568	437.6 (9)

a These ten papers were all published on N Engl J Med.

### Journals

A total of 496 journals published original articles on lung cancer immunotherapy. Among them, *Lung Cancer* (150 papers), *Thoracic Cancer* (138 papers), and *Clinical Lung Cancer* (97 papers) were the three main journals with the most articles (**Figure 2B**). Among the top 10 productive journals, the *Journal of Thoracic Oncology* had the highest average number of citations per article (79.47), the average number of citations per article per year (18.80) and IF (20.121), which indicated that it was not only productive but also influential (**Table 2**).

**Table 2:** The top 10 productive journals in lung cancer immunotherapy from 2010 to 2022.

Journals	Paper number	Total citation	Citation per paper	Citation per paper per year^a^	H-index	G-index	IF (2021)
Lung Cancer	150	4543	30.29	9.07	36	60	6.081
Thorac. Cancer	138	652	4.72	2.84	14	4	3.223
Clin. Lung Cancer	97	1427	14.71	5.49	19	34	4.840
Front. Oncol.	90	312	3.47	2.63	9	12	5.738
Cancer Immunol. Immunother.	80	1654	20.68	8.47	22	39	6.630
J. Immunother. Cancer	74	1575	21.28	8.61	22	37	12.469
Transl. Lung Cancer Res.	68	296	4.35	2.79	9	14	4.726
Cancers	61	417	6.84	4.23	13	18	6.575
J. Thorac. Oncol.	60	4768	79.47	18.80	40	60	20.121
OncoImmunology	49	1293	26.39	6.69	19	35	7.723

a Papers published in 2022 were not included for calculating citation per paper per year.

In particular, the top 10 journals with the highest citations per paper per year differed markedly from the most productive journals (**Figure 2C** and **Table 3**). Among them, the *Annals of Oncology* was the most productive (40 papers). Among all journals, the *N Engl J Med* had the highest total number of citations (31,845), average citation per paper (2,449.62), average citations per paper per year (512.22), and local citations (citation number in the current dataset) (8266), which were much higher than others. *N Engl J Med* only published 13 articles on lung cancer immunotherapy, but these articles accounted for 26.00% citations of all articles in this area. Furthermore, *Journal of Clinical Oncology* (*J Clin Oncol*) had high number of local citations (5,272), which indicated that it was highly influential on lung cancer immunotherapy.

**Table 3:** The top 10 journals with highest citations per paper per year in lung cancer immunotherapy from 2010 to 2022^a^.

Journals	Paper number	Top paper number	Top paper rate	Total citation	Citation per paper	Citation per paper per year^b^	Local citation^c^	IF (2021)
N Engl J Med	13	12	92.31%	31845	2449.62	512.22	8266	176.079
Lancet	7	5	71.43%	9293	1327.57	290.55	2475	202.731
Cancer Cell	3	2	66.67%	941	313.67	114.86	536	38.585
Nat. Med.	6	2	33.33%	1043	173.83	87.33	913	87.241
Lancet Oncol.	24	15	62.50%	7948	331.17	80.08	2218	54.433
Cancer Discov.	6	3	50.00%	1598	266.33	70.19	709	38.272
J Clin Oncol	29	16	55.17%	6926	238.83	66.69	5272	50.717
Lancet Resp. Med.	5	1	20.00%	706	141.20	53.74	140	102.642
JAMA Oncol.	24	9	37.50%	4118	171.58	52.09	1399	33.006
Ann. Oncol.	40	8	20.00%	4668	116.70	33.38	2744	51.769

a Only journals with more than one paper were included.

b Papers published in 2022 were not included for calculating citation per paper per year.

c Citation number in the current dataset (papers in lung cancer immunotherapy from 2010 to 2022).

**Figure 3:**
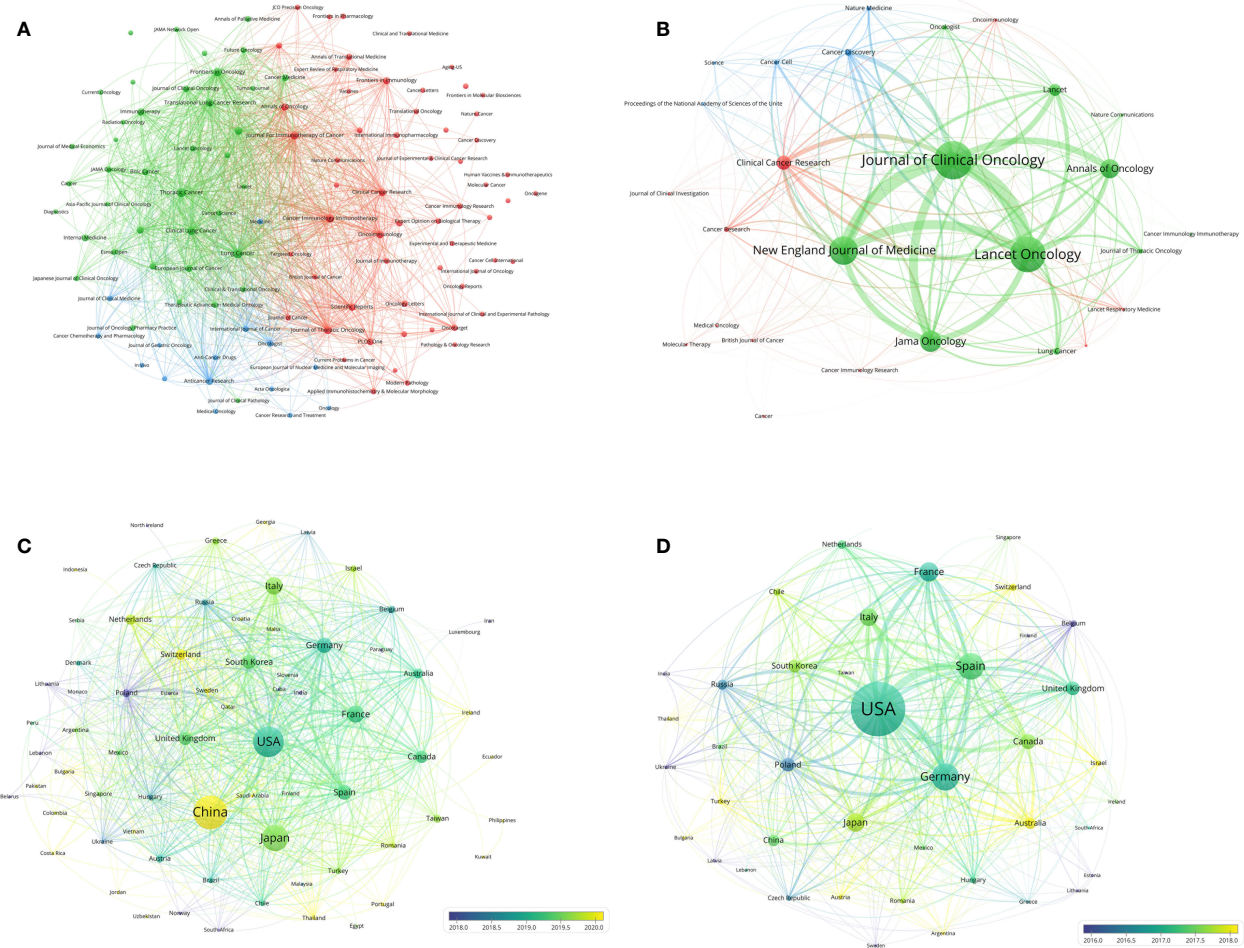
**(A)** Bibliographic coupling of journals with at least five papers related to lung cancer immunotherapy. **(B)** Bibliographic coupling of journals with top papers related to lung cancer immunotherapy. **(C)** Network visualization of countries with papers related to lung cancer immunotherapy. **(D)** Network visualization of countries with top papers related to lung cancer immunotherapy. The circle size represents the number of papers. The breadth of the curves represents the connection strength. The journals in the same color are of similar research areas.

A dual map overlay showed the distribution of academic discipline and the citation relationship of journals related to lung cancer immunotherapy (**Figure 2D**). In the citation relationship indicated by the colored curve, the citing journals are on the left and the cited journals are on the right. This map revealed three primary citation relationship pathways, meaning that papers on molecular/biology/genetics were primarily cited by papers on molecular/biology/immunology and medicine/medical/clinical studies, while papers in health/nursing/medicine were primarily cited by papers in medicine/medical/clinical. The bibliographic coupling network of journals related to lung cancer immunotherapy was conducted (**Figure 3A**).

The authors identified 111 journals with a 5–year IF > 5 published at least two papers on lung cancer immunotherapy. The correlation test showed that the correlation coefficients between average citation per paper per year and (1) 5–year IF and (2) JCI were 0.893 and 0.887, respectively. Therefore, papers published in highly indexed journals are more likely to be highly cited. However, the authors found that although some journals had high IF, the papers published in these journals were not highly cited. For example, *Sci. Adv.* (5–year IF=16.895) had an average citation per paper per year of only 0.923.

The 100 top papers on lung cancer immunotherapy were published in 27 journals (**Supplementary Table S3**). The bibliographic coupling network of these journals was conducted (**Figure 3B**). *J Clin Oncol* (16 papers), *Lancet Oncol.* (15 papers), and *N Engl J Med* (12 papers) were the top three journals with the highest number of papers. The top paper rates (TPR) of the 27 journals were calculated. For journals that published at least two top papers, *N Engl J Med* had the highest TPR (92.31%), followed by *Lancet* (71.43%), *Cancer Cell* (66.67%), *Lancet Oncology* (62.5%) and *J Clin Oncol* (55.17%). The articles on lung cancer immunotherapy published in these journals are highly likely to be top papers. Among the 27 journals, 22 with a TPR >5% were considered the major journals on lung cancer immunotherapy. Since 2020, a total of 110 articles have been published in the major journals (**Supplementary Table S4**). The three major journals with the most publications between 2020 and 1 July 2022 were *Annals of Oncology* (16 papers), *Clinical Cancer Research* (12 papers), and *J Clin Oncol* (12 papers).

### Countries/regions

**Figure 4:**
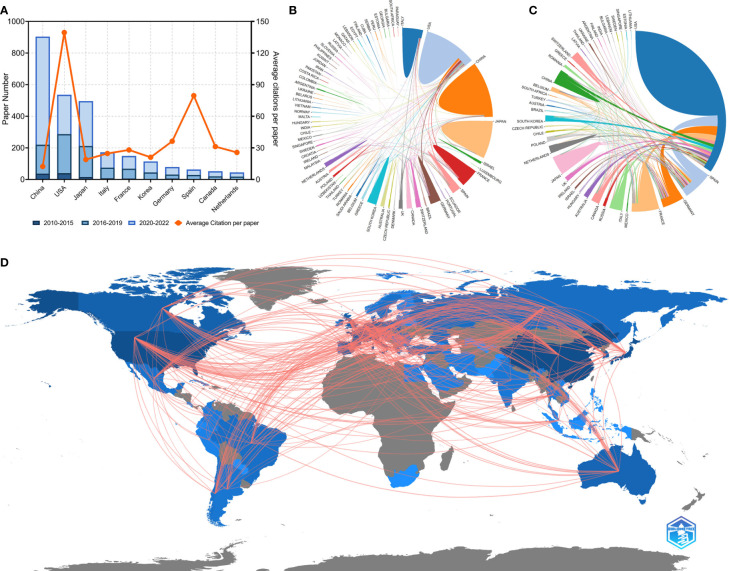
**(A)** Paper number and average citations of corresponding authors’ countries. MCP, multiple-country publications; SCP, single-country publications. **(B)** Network mapping of international collaboration base on 2941 papers related to lung cancer immunotherapy. **(C)** Network mapping of international collaboration base on 100 top papers related to lung cancer immunotherapy. **(D)** Visualization world map of publications and collaboration relationship.

Researchers from 75 countries/regions contributed to the 2,941 original articles on lung cancer immunotherapy. A network visualization map presented the collaboration relationship and the average publication year of the countries/regions (**Figure 3C**). However, the corresponding authors only represent 51 of the countries/regions. The corresponding authors from China contributed the most publications (904 papers), followed by the corresponding authors from the United States (536 papers) and Japan (496 papers) (**Table 4** and **Figure 4A**). However, papers by corresponding authors from the United States were cited as high as 74,751 times, with an average citation per paper of 139.46, which was much higher than in other countries/regions. Most studies were conducted by authors from single countries. International collaboration was more common in North American or European countries than in Asian countries. The rate of multiple–country papers in Japan (3.42%) was the lowest among the most productive countries. A chordal graph and a collaborative network world map showed the collaboration between countries/regions (**Figures 4B, D**). The United States collaborated with most countries/regions in this research area. Most studies supported by developing countries/regions were published more recently than those of developed countries.

**Table 4:** The top 10 productive countries of corresponding authors of papers in lung cancer immunotherapy from 2010 to 2022.

Countries	Paper number	Percentage (N/2941)	Multiple-country paper rate^b^	Total citation	Citation per paper	Top paper number^a^	Multiple-country top-paper rate
China	904	30.74%	12.39%	11127	12.31	5	20.0%
USA	536	18.23%	33.02%	74751	139.46	61	65.6%
Japan	496	16.87%	3.43%	9383	18.92	7	14.3%
Italy	172	5.85%	21.51%	4240	24.65	5	60.0%
France	149	5.07%	28.86%	4177	28.03	4	100.0%
Korea	114	3.88%	9.65%	2403	21.08	1	100.0%
Germany	79	2.69%	43.04%	2866	36.28	4	100.0%
Spain	63	2.14%	42.86%	5011	79.54	7	100.0%
Canada	52	1.77%	40.38%	1623	31.21	2	100.0%
Netherlands	45	1.53%	37.78%	1155	25.67	2	50.0%

a Besides the countries mentioned above, corresponding authors from Belgium and Switzerland contributed one top-paper each.

b Percentage of multiple-country top papers among all papers of a country.

The 100 top papers were published by authors from 42 countries/regions and corresponding authors from 12 countries/regions. A network visualization map presented the collaboration relationship and average publication year of the countries/regions with the top papers (**Figure 3D**). The corresponding authors of most of the top papers (61 papers) represented the United States. International collaboration was more common in the top papers than in all the papers on lung cancer immunotherapy (**Figure 4C**). International collaboration, however, remained rare in China and Japan, which had only one top paper with foreign authors.

### Institutions

**Figure 5:**
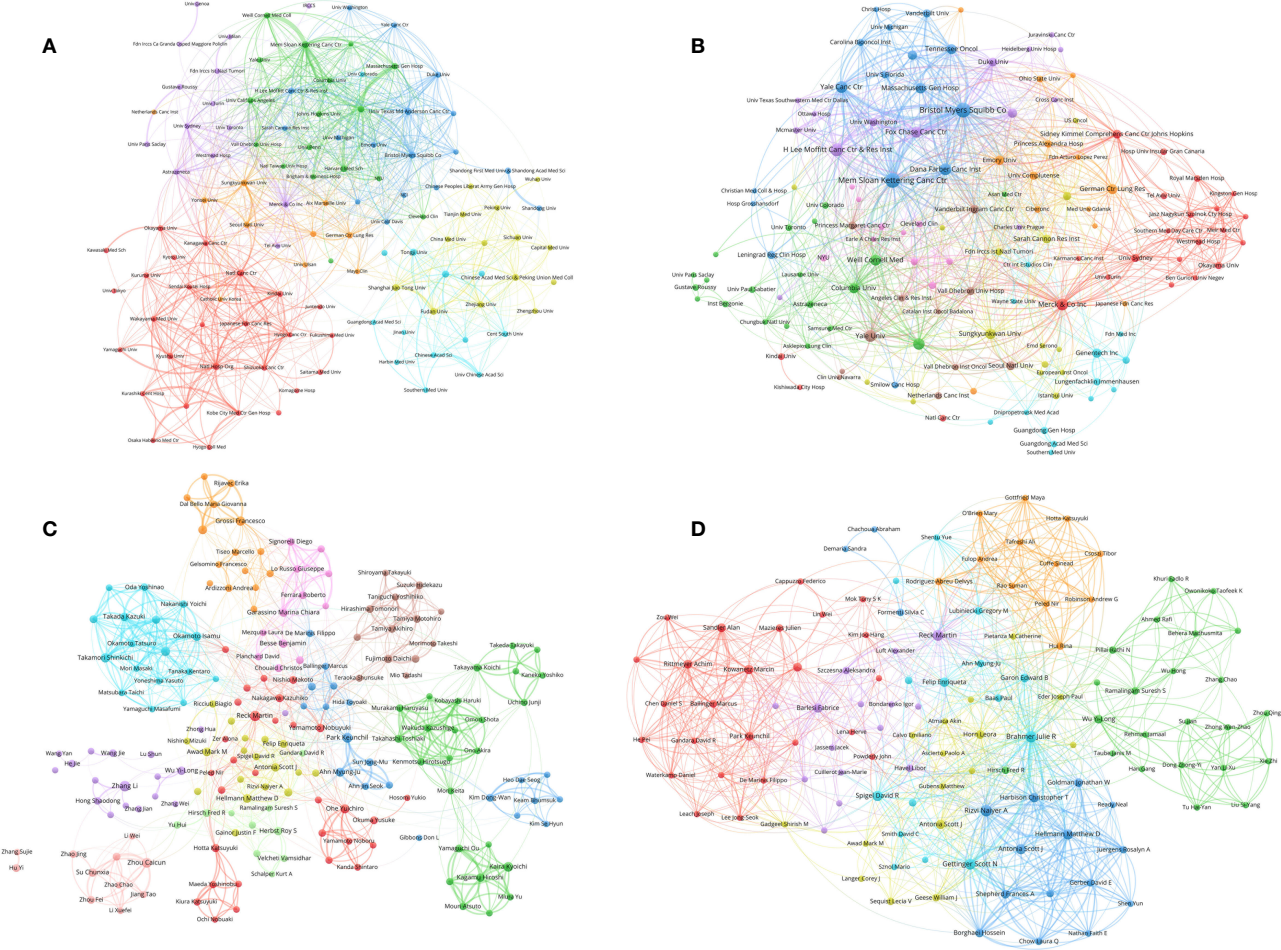
**(A)** Network visualization of institutions with at least 15 papers related to lung cancer immunotherapy. **(B)** Network visualization of institutions with at least 2 top papers related to lung cancer immunotherapy. **(C)** Network visualization of authors with at least 10 papers related to lung cancer immunotherapy. **(D)** Network visualization of authors with at least 2 top papers related to lung cancer immunotherapy. The circle size represents the number of papers. The breadth of the curves represents the connection strength. The institutions in the same color have stronger collaboration with each other.

The authors of the 2,941 papers represented 4,296 institutions. The University of Texas MD Anderson Cancer Center contributed most articles (224 papers) among institutions (**Table 5**). Seven of the 10 most productive institutions were in China and the other 3 were in the United States. A collaboration network and a cluster analysis of the institutions were conducted (**Figure 5A**). Most institutions preferred domestic collaboration over international collaboration. International collaboration was common between the institutions with the strongest research strength in their countries.

**Table 5:** The top 10 institutions with the most papers or top papers on lung cancer immunotherapy from 2010 to 2022.

Institutions	Country	Paper number^a^	Percentage (N/2941, %)	Top paper number	Top paper rate	Top paper number rank
Univ Texas Md Anderson Canc Ctr	USA	224	7.62%	14	6.25%	8
Tongji Univ	China	204	6.94%	0	0.00%	N/A
Fudan Univ	China	193	6.56%	0	0.00%	N/A
Sun Yat Sen Univ	China	190	6.46%	1	0.53%	214
Shanghai Jiao Tong Univ	China	182	6.19%	2	1.10%	124
Sichuan Univ	China	153	5.20%	0	0.00%	N/A
Chinese Acad Med Sci and Peking Union Med Coll	China	147	5.00%	0	0.00%	N/A
Mem Sloan Kettering Canc Ctr	USA	138	4.69%	48	34.78%	1
Natl Canc Ctr	China	116	3.94%	6	5.17%	36
Dana Farber Canc Inst	USA	115	3.91%	16	13.91%	5
H Lee Moffitt Canc Ctr and Res Inst	USA	102	3.47%	24	23.53%	3
Johns Hopkins Univ	USA	93	3.16%	19	20.43%	4
Yale Univ	USA	78	2.65%	33	42.31%	2
Univ Calif Los Angeles	USA	68	2.31%	15	22.06%	7
Sarah Cannon Res Inst	UK	47	1.60%	16	34.04%	5
German Ctr Lung Res	Germany	35	1.19%	14	40.00%	8

a All papers were included, without limitation of corresponding author’s institutions. NA, Not applicable.

A total of 583 institutions contributed to top papers. The three leading productive institutions of the top papers were the Memorial Sloan–Kettering Cancer Center (48 papers), Yale University (33 papers), and the H Lee Moffitt Cancer Center and Research Institution (24 papers). In particular, Yale University had the highest TPR (42.31%). Although some institutions in China contributed to a large number of papers, their number of top papers was low. A collaboration network and cluster analysis of the institutions with top papers was performed (**Figure 5B**). Compared to the clusters in **Figure 5A**, the clusters of the institutions with top papers had more obscure boundaries. Collaboration between institutions with top papers was common and less restricted by geographical factors.

### Authors

A total of 15,017 researchers contributed to the 2,941 original articles on lung cancer immunotherapy. Reck M was the most cited author in this area (44 papers, 22,380 citations), followed by Hellmann MD (35 papers, 18,839 citations) and Paz–Ares L (25 papers, 18,588 citations) (**Table 6**). Notably, although only published 6 papers in this area, Lubiniecki GM was the sixth cited author (15,257 citations). Reck M and Brahmer JR published their first papers in 2012 and 2013, respectively, which indicated that they joined this research area early. Other eight most cited authors published their first papers in 2015. A collaboration network and clustering analysis of the coauthors was conducted (**Figure 5C**). The authors of China and Japan preferred to establish stable collaborations with researchers in their own countries rather than with foreign researchers.

**Table 6:** Top 10 most cited authors related to lung cancer immunotherapy from 2010 to 2022.

Name	Paper number	Total citation	H-index	Average citations per paper	Articles fractionalized^a^	Top paper number	Top paper rate	First publication year
Reck M	44	22380	29	508.64	4.52	17	38.64%	2012
Hellmann MD	35	18839	32	538.26	2.38	17	48.57%	2015
Paz-Ares L	25	18588	19	743.52	1.88	11	44.00%	2015
Horn L	25	17583	24	703.32	1.66	13	52.00%	2015
Garon EB	22	17190	19	781.36	1.8	8	36.36%	2015
Lubiniecki GM	6	15257	6	2542.83	0.34	5	83.33%	2015
Brahmer JR	21	15116	19	719.81	1.91	6	28.57%	2013
Felip E	24	13763	21	573.46	1.27	10	41.67%	2015
Rizvi NA	17	13704	16	806.12	1.42	12	70.59%	2015
Spigel DR	17	12660	15	744.71	1.31	7	41.18%	2015

a Articles Fractionalized = paper number/total number of authors of the papers.

The analysis of corresponding authors might highlight the main contributors to the articles. A total of 2,005 corresponding authors were identified. As corresponding author, Zhang L contributed to most papers (16 papers), but these papers were only cited 450 times (**Table 7**). Hellmann MD was corresponding author for 14 papers (5,394 citations) and Reck M was corresponding author for 12 papers (2,093 citations). Brahmer JR was the most cited corresponding author with 4 papers (9,966 citations). Notably, Hellmann MD was the only one who was both one of the ten most productive and cited corresponding authors.

**Table 7:** The top 10 productive and cited corresponding authors in lung cancer immunotherapy from 2010 to 2022.

Most productive corresponding author	Paper number	Total citation	Average citations per paper	Top paper number	Most cited corresponding author	Paper number	Total citation	Average citations per paper	Top paper number
Zhang L	16	450	28.13	0	Brahmer JR	4	9966	2491.50	2
Hellmann MD	14	5394	385.29	7	Borghaei H	5	6057	1211.40	1
Reck M	12	2093	174.42	4	Hellmann MD	14	5394	385.29	7
Fujimoto D	12	551	45.92	1	Chan TA	1	4908	4908.00	1
Wu YL	12	793	66.08	2	Garon EB	8	4280	535.00	2
Takada K	11	171	15.55	0	Herbst RS	3	3441	1147.00	1
Yamada T	11	125	11.36	0	Gandhi L	1	2827	2827.00	1
Awad MM	10	440	44.00	0	Gandara DR	1	2647	2647.00	1
Zhou CC	12	363	30.25	0	Paz-Ares L	6	2483	413.83	3
Kaira K	9	128	14.22	0	Rizvi NA	5	2103	420.60	4

A total of 1,498 authors contributed to the 100 top papers. Reck M and Hellmann MD was the most productive authors of the top papers (17 papers each), followed by Horn L (13 papers). A collaboration network and clustering analysis of the coauthors of the top papers was conducted (**Figure 5D**). International collaboration between authors was common and some close partnerships between Asian researchers and American or European researchers were revealed. Fourteen corresponding authors contributed to at least two top papers (**Supplementary Table S5**). Hellmann MD was the most productive corresponding author (7 papers), followed by Rizvi NA and Reck M (4 papers each).

### Keywords

**Figure 6:**
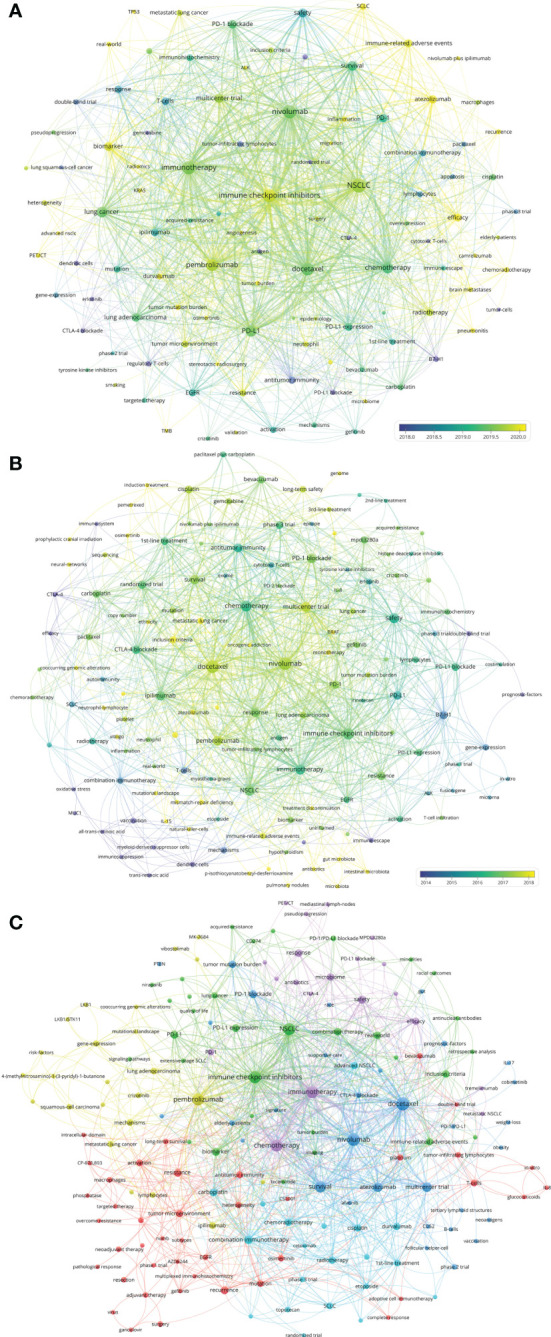
**(A)** Network visualization of keywords that occurred at least 15 times in the papers. **(B)** Network visualization of keywords the top papers. **(C)** Network visualization of keywords in papers published in major journals between 2020 and 2022. The circle size represents the number of papers. The breadth of the curves represents the connection strength.

Based on author–chose keywords and keyword–plus identified by WoSCC, the hot keywords were analyzed in multiple dimensions. The trends and variation of keyword occurrence frequencies in lung cancer immunotherapy from 2010 to 2022 were analyzed and visualized (**Supplementary Figure S4**). “Tumor microenvironment”, “radiotherapy”, “biomarker”, and “immune–related adverse events (irAEs)” are recently rising keywords. The top 25 keywords with the strongest citation bursts were identified (**Supplementary Figure S5**). Vaccine and adoptive cell immunotherapy (ACT) used to be research hotspots, and ICIs became new hotspots since 2015. The co–occurrence and citation network of the keywords of the 2,941 papers was conducted (**Figure 6A**). The top–keywords included “nivolumab”, “pembrolizumab”, “docetaxel”, “PD–L1”, “epidermal growth factor receptor (EGFR)”, “survival”, and “safety”. Recently occurred keywords included “SCLC”, “irAEs”, “anaplastic lymphoma kinase (ALK)”, “biomarker”, “atezolizumab”, “duvalumab”, “radiomics”, “tumor mutation burden (TMB)”, “tumor burden”, “chemoradiotherapy”, and “microbiome”.

The co–occurrence and citation network of the keywords of the 100 top papers was conducted (**Figure 6B**). The newly utilized keywords included “bevacizumab”, “acquired–resistance”, “BRAF”, “monotherapy”, “vitiligo”, “mutational–landscape”, “mismatch–repair deficiency”, “treatment discontinuation”, “tumor–infiltrating lymphocytes (TILs)”, and “antibiotics”. The keyword co–occurrence and citation network of the 110 recently published papers in major journals was conducted (**Figure 6C**). The keywords which were different from previous analysis included “heterogeneity”, “neoantigens”, “adjuvant therapy”, “neoadjuvant therapy”, “targeted therapy”, “overcome resistance”, “elderly–patients”, “niraparib”, and some new molecular targets.

### Research trends

**Figure 7:**
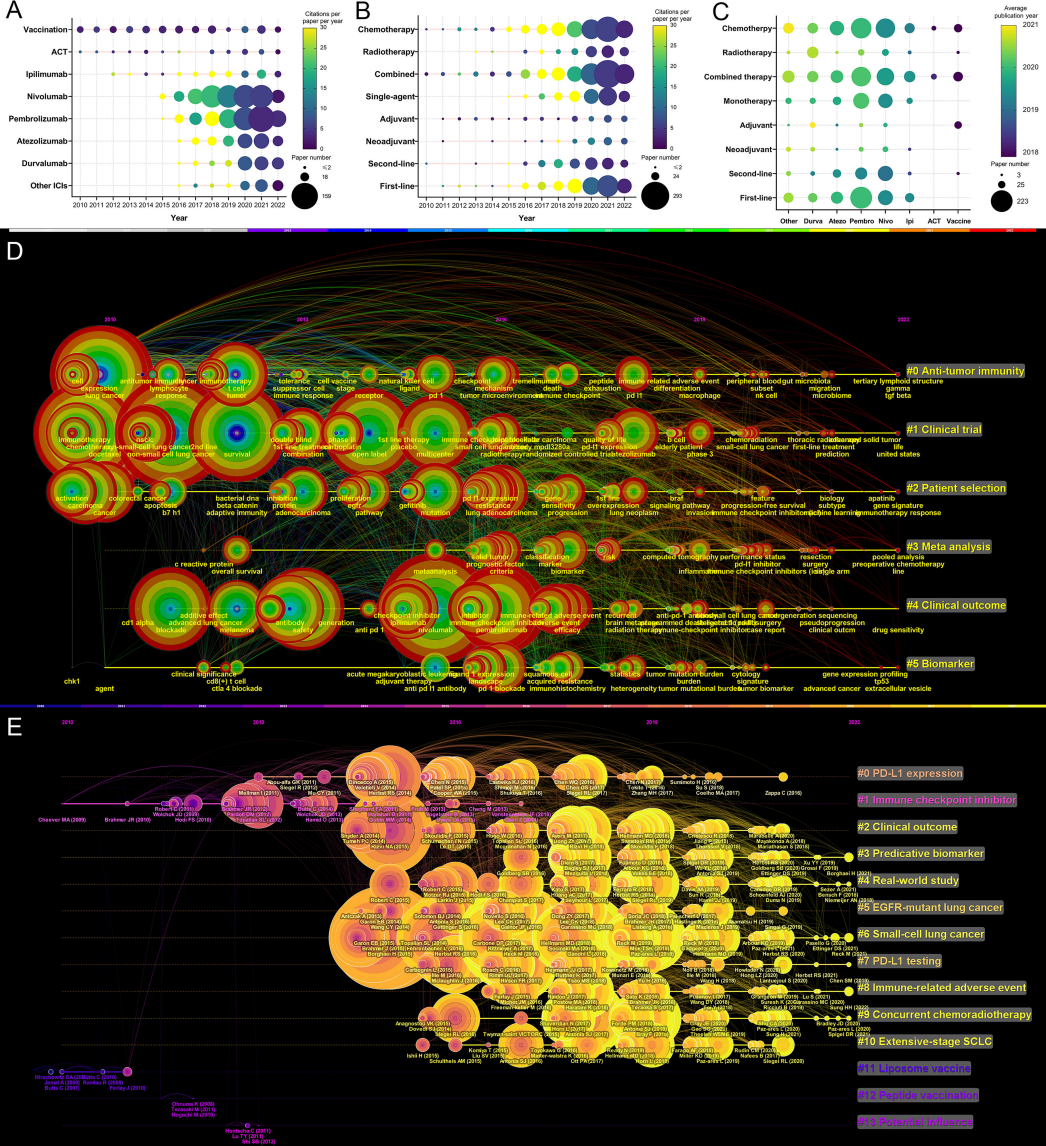
**(A)** Publication number and citations per paper per year of different therapies/drugs. The node size represents the paper number and the color represents the average citations per paper. **(B)** Publication number and citations per paper per year of different treatments. The node size represents the paper number and the color represents the average citations per paper. **(C)** The publication number and average publication year of therapies/drugs for different treatments. The node size represents the paper number and the color represents the average publication year. **(D)** The timeline view for co-cited keywords related to lung cancer immunotherapy. The node size represents the citation number of the reference. The curves between the nodes indicated co-citation relationships. **(E)** The timeline view for co-cited references related to lung cancer immunotherapy. The node size represents the citation number of the reference. The curves between the nodes indicated co-citation relationships. Yellow nodes represent new papers and red nodes represent old ones.

The number of publications and the total number of citations per paper per year of article publication of the eight immunotherapies for lung cancer are presented in **Figure 7A**. Between 2010 and 2022, publications related to vaccines or ACT varied slightly. The number of publications related to ipilimumab (an anticytotoxic T lymphocyte–associated protein 4 [CTLA–4] antibody) has grown slowly since 2012. The number of publications for the two most well–known anti–PD–1 antibodies, nivolumab and pembrolizumab, has increased markedly since 2015. The research on anti–PD–L1 antibodies was reported a little later. In 2016, the first articles on atezolizumab and durvalumab for lung cancer were published.

The variation in the treatment pattern was analyzed and presented in **Figure 7B**. In recent years, reports related to radiotherapy, single–agent therapy, and first–line therapy have gradually increased. The relationship between the treatment pattern and the immunotherapy modalities was analyzed and is presented in **Figure 7C**. Among immunotherapies, nivolumab and pembrolizumab had the most related publications. In general, articles on pembrolizumab were slightly more recent than the articles on nivolumab, and pembrolizumab had more related articles on first–line treatment than nivolumab. Compared to first– and second–line treatment, there were fewer articles on adjuvant or neoadjuvant treatment.

A timeline view for the variation of co–cited keywords related to lung cancer immunotherapy is presented in **Figure 7D**. Keywords were classified into six clusters. Recent research hotspots included “gut microbiota”, “tertiary lymphoid structure”, “prediction”, “gene signature”, “sequencing”, “extracellular vesicle”. A timeline view of the co–cited reference variation related to lung cancer immunotherapy is presented in **Figure 7E**. The references were classified into 14 clusters. The topics with large yellow nodes, which represented many recent articles, were research hotspots. Recent hot topics included “clinical outcome”, “predictive biomarker”, “real–world study”, “SCLC”, “irAE”, and “concurrent chemoradiotherapy (CCRT)”.

## Discussion

Given its high global disease burden, lung cancer has always been a highly regarded research area. The clinical application of tyrosine kinase inhibitors (TKIs) greatly improved the prognosis of patients with EGFR or ALK gene altered NSCLC (1). In recent years, even for Kirsten rat sarcoma (KRAS) gene alterations, used to considered “un–targetable”, new TKIs have emerged (16). However, until the advent of ICIs, the outcomes of patients with SCLC or wild–type driver gene NSCLC remained unsatisfactory. Ipilimumab, an anti–CTLA–4 antibody, improved the prognosis of these patients combined with chemotherapy (17, 18). Subsequently, anti–PD–(L)1 therapy showed even greater efficacy and safety (19, 20). In recent years, anti–PD–(L)1 therapy has become a standard treatment for a large portion of patients with lung cancer. Furthermore, other immunotherapies such as dual–ICIs, cancer vaccine, ACT and T–cell immunoreceptor with Ig and ITIM domain (TIGIT) blockade also showed clinical value in selected lung cancer patients (21–24). ​Currently, Real–world studies have demonstrated the improvement of OS with the evolution of anticancer pharmacological treatments over the past decade (25). However, primary and acquired resistance, as well as irAEs, limit further improvement in prognosis.

### Anti–PD–(L)1 for NSCLC

**Figure 8:**
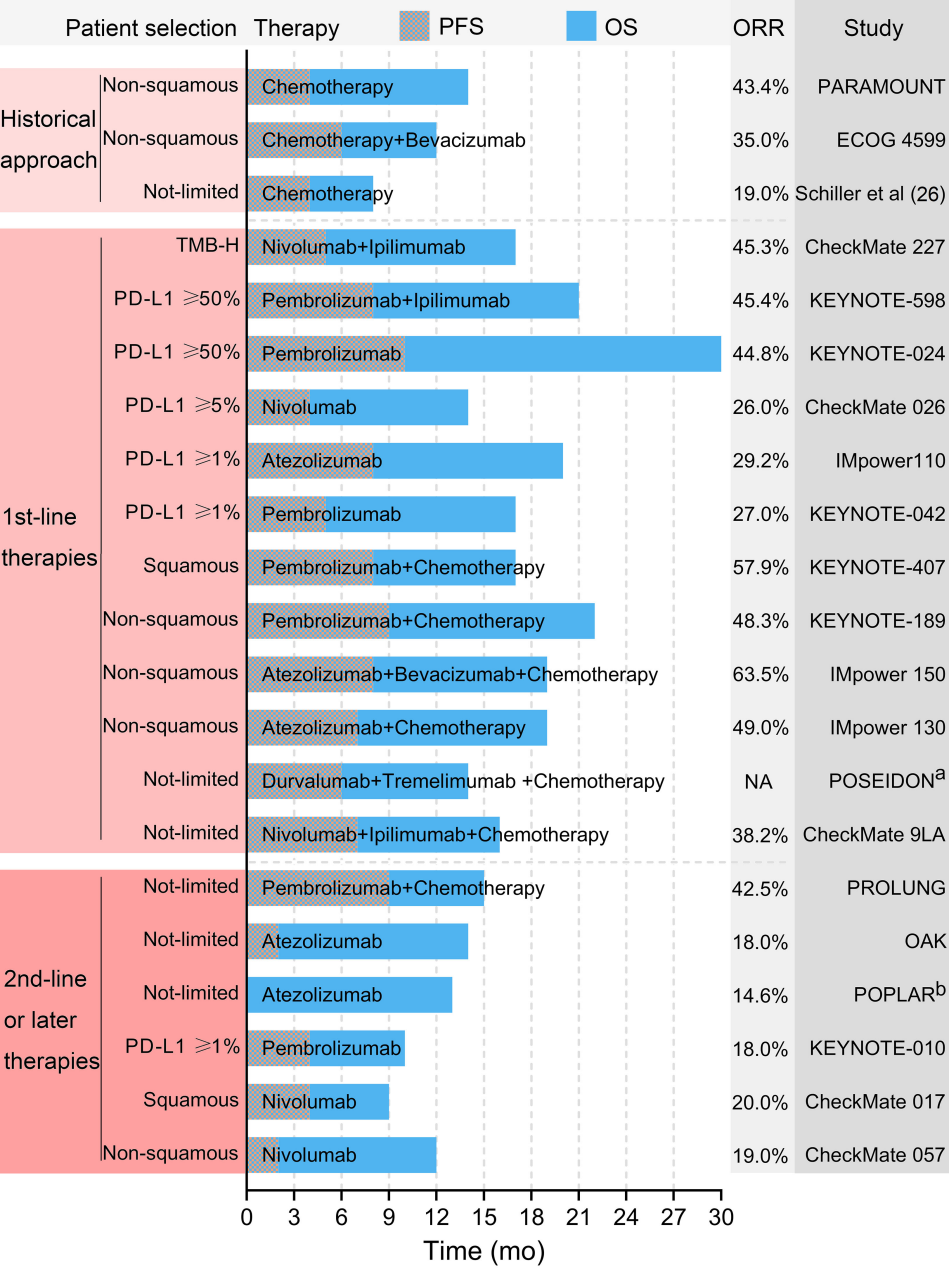
Selected outcomes reported by influential studies. ^a^ The POSEIDON trial did not report the ORR. ^b^ The POPLAR trial did not report the PFS. PFS, progression-free survival. OS, overall survival. ORR, objectively response rate. TMB-H, high tumor mutational burden.

The historical approach to metastatic NSCLC involved chemotherapy, with an overall survival time (OS) of as short as 8–14 months (26–28). In recent years, the OS of patients with metastatic NSCLC with aberrations of the EGFR/ALK gene has been extended to over 3 years with targeted therapy (29, 30). For patients with NSCLC without targetable gene alteration, immunotherapy was a significant choice. Patients with high expression of PD–L1 or high TMB were more likely to benefit from anti–PD–(L)1 therapy (31, 32). **Figure 8** summarizes the outcomes reported by influential studies on advanced NSCLC. Furthermore, adjuvant/neoadjuvant anti–PD–(L)1 therapy has been a research hotspot, which could further reduce the recurrent risk of patients with resectable NSCLC.

### Anti–PD–(L)1 for previously treated advanced NSCLC

PD–(L)1 ICIs were initially evaluated as 2^nd^–line or subsequent treatment for advanced NSCLC. The pioneer phase 3 randomized trials were CheckMate 017 and CheckMate 057, which evaluated nivolumab (an anti–PD–1 antibody) for previously treated squamous and non–squamous NSCLC, respectively, and published their results in 2015 (13, 33). The results showed that nivolumab was more effective and safer than docetaxel regardless of PD–L1 expression in tumor cells, and the expression of PD–L1 was not a prognostic factor in patients treated with nivolumab (13, 33). Subsequently, pembrolizumab (an anti–PD–1 antibody) and atezolizumab (an anti–PD–L1 antibody) were shown, respectively, to be appropriate 2^nd^–line treatments for advanced NSCLC by the KEYNOTE–010 and POPLAR trials (34, 35). In contrast to previous results, these trials revealed some prognostic or predictive factors in patients treated with ICIs. The KEYNOTE–010 trial only enrolled patients with PD–L1 expressed NSCLC, and found that patients with a tumor proportion score (TPS) for PD–L1 of at least 50% achieved a superior outcome (34). The POPLAR phase 2 trial suggested that although PD–L1 negative patients also benefit from atezolizumab, patients with PD–L1 expressed tumor cells or TIL achieved longer OS (35). The phase 3 OAK trial published in 2017 reported results comparable to those of the POPLAR trial (36).

Although ICIs prolonged the OS of patients, the ORR of anti–PD–(L)1 therapy for previously treated NSCLC was only 14–20% (13, 33–36). Therefore, the combination of anti–PD–(L)1 and anti–CTLA–4 ICIs was evaluated. A phase 1 trial supported that dual–ICIs therapy with specific dose schedules resulted in manageable toxicity and responses regardless of PD–L1 status (37). However, phase 3 trials (S1400I and ARCTIC) did not support the superiority of dual–ICIs over single agent anti–PD–(L)1 therapy for previously treated patients with advanced NSCLC (38, 39). A recently published trial evaluated dual–ICIs plus palliative radiotherapy for patients with anti–PD–(L)1–resistant metastatic NSCLC. However, radiotherapy did not improve efficacy and this trial was terminated after interim analysis (40). Some trials evaluated ICI plus chemotherapy for previously treated patients with advanced NSCLC to further improve response rate. The phase 2 PROLUNG trial demonstrated that pembrolizumab plus docetaxel resulted in a higher ORR (42.5% vs. 15.8%) and a longer progression–free survival (PFS) (9.5 months vs. 3.9 months) than docetaxel alone for patients with advanced previously treated NSCLC (41). A phase 2 trial (PEMBRO–RT) reported additional stereotactic ablative radiotherapy (SABR) before pembrolizumab improved ORR (36% vs. 18%, *P*=0.07) for previously treated patients with metastatic NSCLC (42). In particular, patients lacking PD–L1 expression achieved a greater improvement in PFS and OS than patients with PD–L1 expression (42).

Patients with driver genes–altered NSCLC had limited treatment choices after resistance of tyrosine kinase inhibitors (TKIs). ICI plus chemotherapy or antiangiogenic therapy was a considerable advancement for these patients. In 2019, a subgroup analysis of patients with EGFR mutations in the IMpower150 trial demonstrated that atezolizumab plus bevacizumab and chemotherapy resulted in better OS compared to bevacizumab plus chemotherapy (43). A retrospective analysis compared pembrolizumab plus chemotherapy or anlotinib with pembrolizumab alone for previously treated EGFR–mutated NSCLC. The combined therapy resulted in a significantly better prognosis than monotherapy (44).

As a portion of the patients achieved a durable response, the optimal duration of ICI therapy needed to be clarified. However, the results of the phase 3b/4 CheckMate 153 trial demonstrated that patients treated with continuous nivolumab therapy achieved significantly longer OS and PFS than patients treated with fixed–duration nivolumab therapy for 1 year (45). A retrospective analysis suggested that discontinuous treatment due to irAE was correlated with shorter OS compared to continuous anti–PD–1 therapy (46). Although discontinuation of treatment in selected patients with melanoma did not alter the duration of the response, it should be cautious to suspend ICI therapy in previously treated patients with NSCLC (47).

Because an increasing number of patients have received anti–PD–(L)1 therapy as 1^st^–line treatment in recent years, it is important to establish 2^nd^–line immunotherapy strategies that could overcome immune–resistance. Some TKIs targeting the tumor microenvironment might synergize with anti–PD–(L)1 therapy. Recently, the LUNG–MAP S1800A phase 2 trial reported that ramucirumab (a CTLA–4/vascular endothelial growth factor [VEGF] inhibitor) plus pembrolizumab achieved improved efficacy (objective response rate [ORR], 28%; median OS, 14.5 months) in patients with NSCLC progressed after anti–PD–(L)1 therapy (48). Similarly, cabozantinb plus atezolizumab and sitravatinib plus nivolumab, respectively, showed antitumor immune activity for immune–failed patients with NSCLC in the COSMIC–021 and MRTX–500 trials (49, 50). Phase 3 trials such as SAPPHIRE are ongoing. In particular, a recently published pooled analysis of five phase 3 trials evaluated the second course of pembrolizumab for patients with progression of the disease after at least 2 years since the end of the first course of pembrolizumab. The results showed that the second course was beneficial to the patients (51). Another study reported that 2^nd^–line anti–PD–1 therapy after 1^st^–line anti–PD–L1 therapy or 2^nd^–line anti–PD–L1 therapy after 1^st^–line anti–PD–1 therapy could be superior to chemotherapy (52).

Anti–PD–(L)1 has become the standard treatment for patients with advanced NSCLC who progress after chemotherapy. However, the ORR remains unsatisfactory. ICI combined with other therapies may further improve prognosis, but more clinical evidence is required, and tolerability should be evaluated. Combined therapy including anti–PD–(L)1 is considerable for patients resistant to TKI with EGFR–mutated NSCLC. Furthermore, studies evaluating novel, optimal, and individual treatment strategies for patients resistant to anti–PD–(L)1 therapy are needed.

### First–line anti–PD–(L)1 therapy for advanced NSCLC

Since 2016, a growing number of influential trials have focused on 1^st^–line anti–PD–(L)1 therapy for advanced NSCLC. The KEYNOTE–024 trial enrolled untreated patients with advanced NSCLC with PD–L1 TPS of at least 50%. Pembrolizumab monotherapy achieved significantly higher ORR (44.8% vs. 27.8%), longer PFS, and less toxicity than chemotherapy (14). In that trial, patients in the chemotherapy group could switch to pembrolizumab after progression, but patients in the pembrolizumab group still achieved longer OS (30.0 vs 14.2 months) (53). The CheckMate 026 trial enrolled patients with NSCLC with PD–L1 TPS of at least 5%. However, 1^st^–line nivolumab therapy did not achieve better efficacy than chemotherapy (54). The IMpower110 trial reported similar results that atezolizumab improved the outcome only in patients with high expression of PD–L1, but not in all patients with expression of PD–L1 (55). In contrast, the KEYNOTE–042 trial reported that 1^st^–line pembrolizumab therapy achieved a better prognosis than chemotherapy for patients with NSCLC in all subgroups (PD–L1 TPS between 1% and 20%, between 20% and 50% and at least 50%) (56).

First–line anti–PD–(L)1 monotherapy showed minor toxicity and improved response in selected patients. Anti–PD–(L)1 combined with other therapies was evaluated to further improve efficacy and expand indications. In 2016, the phase 2 KEYNOTE–021 trial first demonstrated that 1^st^–line pembrolizumab plus chemotherapy achieved superior efficacy over chemotherapy (ORR, 55% vs. 29%) for patients with non–squamous NSCLC with/without PD–L1 expression (57). This result was confirmed by the KEYNOTE–189 phase 3 trial (58). Similar results were found in the IMpower 130 trial, which compared atezolizumab plus chemotherapy with chemotherapy (ORR, 49% vs. 32%) as 1^st^–line treatment for non–squamous NSCLC (59). Furthermore, the IMpower 150 trial further showed that the addition of atezolizumab to bevacizumab plus chemotherapy as the 1^st^–line treatment further improved ORR (63.5%) in patients with non–squamous NSCLC; however, the reported OS and PFS between IMpower 130 and IMpower 150 were similar (60). Recently, the phase 2 CAPAP lung trial reported that 1^st^–line camrelizumab (an anti–PD–1 antibody) combined with apatinib and albumin paclitaxel produced encouraging efficacy (ORR, 73.1%) and acceptable safety for advanced non–squamous NSCLC (61). Anti–PD–(L)1 plus chemotherapy was evaluated for previously untreated squamous NSCLC, and the KEYNOTE–407 trial demonstrated the superiority of combined therapy (ORR, 57.9% vs. 38.4%) including pembrolizumab (62).

Nivolumab plus ipilimumab (N+I) was the most common combination of dual–ICIs as the 1^st^–line treatment for NSCLC. The CheckMate 227 trial only enrolled patients with high TMB, and patients treated with N+I also achieved better efficacy (ORR, 45.3% vs. 26.9%) (63). The CheckMate 277 trial suggested that N+I resulted in better efficacy (ORR, 35.9% vs. 30.0%) and comparable safety to chemotherapy (21). Furthermore, CheckMate 9LA demonstrated that N+I plus two cycles of chemotherapy achieved a higher ORR (38.2%) (15). Similarly, the POSEIDON trial demonstrated that durvalumab plus tremelimumab combined with chemotherapy was superior to chemotherapy alone as 1^st^–line treatment for metastatic NSCLC (64). However, the KEYNOTE–598 trial did not support the improvement in efficacy of adding ipilimumab to pembrolizumab, but increased toxicity, as the 1^st^–line treatment for patients with NSCLC with PD–L1 TPS of at least 50% (65). Recently, a randomized trial (SQUINT) demonstrated that 1^st^–line N+I achieved similar efficacy and superior safety compared to nivolumab plus chemotherapy for advanced squamous NSCLC (66).

Anti–PD–(L)1 plus chemotherapy is suitable for previously untreated advanced NSCLC without PD–L1 expression and driver gene alteration. However, the optimal therapeutic modality for PD–L1 positive NSCLC remains uncertain. In 2021, the US Food and Drug Administration reported a pooled analysis of anti–PD–(L)1 plus chemotherapy vs. anti–PD–(L)1 alone as 1^st^–line treatment for advanced NSCLC with PD–L1 TPS 1–49% (67). Combined therapy showed better OS (21.4 months vs. 14.5 months) and PFS (7.7 months vs. 4.2 months) than anti–PD–(L)1 monotherapy in these patients. Furthermore, patients ≥75 years old experienced a similar prognosis in both groups (67). A recently published pooled analysis of NSCLC with PD–L1 TPS ≥50% demonstrated the superiority of combined therapy (OS, 25.0 months vs. 20.9 months; PFS, (9.6 months vs. 7.1 months) (68). Similarly, patients ≥75 years old may not benefit from combined therapy (68). In the 2022 World Conference on Lung Cancer (WCLC 2022), Dong et al. reported that adding radiotherapy to first–line anti–PD–1 plus chemotherapy for advanced NSCLC significantly improved OS and PFS (69).

Anti–PD–(L)1 plus chemotherapy has been established as the standard 1^st^–line treatment for the most advanced patients with NSCLC with no targetable driver gene mutation. Some patients may benefit from combination therapy that includes antiangiogenic or anti–CTLA–4 therapy, but more clinical evidence is warranted. The optimal treatment for older patients or patients with poor tolerance may be anti–PD–(L)1 monotherapy. However, the therapeutic strategy for other special patients, such as patients with KRAS gene alterations or brain metastases, remains unspecified. Future studies are warranted to develop next–generation therapies, establish robust predictive models, and determine individual treatment patterns for specific subgroups of patients.

### Novel immunotherapy for advanced NSCLC

The efficacy of current immunotherapy–based treatment remains unsatisfactory and the treatment option for patients with anti–PD–(L)1–resistant NSCLC is limited. Currently, multiple novel immunotherapies targeting different signal pathways are being developed, including lymphocyte activation gene–3 (LAG–3), Janus kinase 1 (JAK1), and T cell immunoreceptors with Ig and ITIM domains (TIGIT) antibodies. Furthermore, bispecific antibodies that could block both PD–(L)1 and VEGF such as KN046, MEDI5752, and AK112 are being evaluated for advanced NSCLC.

LAG–3 blockade (relatlimab) has been shown to be beneficial when combined with nivolumab for advanced melanoma (70). Recently, the phase 2b TACTI–003 trial reported that eftilagimod alpha (a soluble LAG–3 protein) plus pembrolizumab achieved favor efficacy (ORR, 38%; PFS, 6.9 months) as 1^st^–line treatment for advanced NSCLC (71). An anti–TIGIT antibody tiragolumab plus atezolizumab was considered superior to atezolizumab monotherapy for previously untreated patients with NSCLC in the CITYSCAPE phase 2 trial (24). However, the recently announced interim results of the SKYSCRAPER–01 phase 3 trial that evaluated tiragolumab plus atezolizumab as 1^st^–line treatment for patients with PD–L1–high metastatic PD–L1 NSCLC did not meet its primary endpoint. Other trials (e.g., KEYVIBE–007, AdvanTIG–302) are ongoing to further evaluate 1^st^–line anti–TIGIT (e.g., vibostolimab, ociperlimab) plus anti–PD–1 therapy for NSCLC. Clinical evidence supporting immunotherapy for other targets, such as interleukin–1β and hematopoietic progenitor kinase 1, is still lacking. In WCLC 2022, a phase 2 trial reported that pembrolizumab plus itacitinib (a JAK1 inhibitor) was effective (ORR, 62%) and safe for metastatic NSCLC with PD–L1 TPS of at least 50% (72). Another phase 2 trial (HUDSON) suggested durvalumab plus ceralasertib (an ATR inhibitor) might be an effective treatment option for patients with NSCLC who failed from chemotherapy and anti–PD–L1 therapy (73).

AK112, a PD–1/VEGF bispecific antibody, plus chemotherapy, showed promising antitumor efficacy for patients who had not previously been treated, failed prior EGFR–TKI, or progressed after anti–PD–(L)1 plus chemotherapy in a recent phase 2 trial (74). Among the patients in the three cohorts, the ORR was reported to be 76.9%, 68.4%, and 40.0%, respectively (74). The preliminary results of trials support the antitumor activity of other bispecific antibodies, but clinical evidence on NSCLC is lacking.

Because combinations of existing drugs can hardly further improve the prognosis and overcome resistance of advanced NSCLC, novel immunotherapies are urgently needed. The main research directions include new targets and bispecific antibodies, but clinical evidence is lacking, and many new approaches have not been successful during clinical trials. In addition, basic research on tumor immune microenvironment may promote the understanding of immune–resistance mechanisms, thus guiding the development of novel approaches.

### Adjuvant anti–PD–(L)1 therapy

Because anti–PD–(L)1 therapy showed encouraging efficacy and manageable toxicity for advanced NSCLC, it is reasonable to evaluate anti–PD–(L)1 therapy as adjuvant treatment after radical surgery or chemoradiotherapy. The most influential phase 3 trial that evaluated adjuvant anti–PD–(L)1 for NSCLC was PACIFIC, which first reported that durvalumab after concurrent chemoradiotherapy improved PFS and OS (75, 76). Recent published 5–year results confirmed the superiority of the results achieved with durvalumab over placebo (median OS, 47.5 vs. 29.1 months; median PFS, 16.9 vs. 5.6 months) (77). In 2020, the LUN 14–179 phase 2 trial suggested that consolidation of pembrolizumab after chemoradiotherapy also improved PFS and OS in patients with stage III NSCLC (78). Another nonrandomized phase 2 trial (KEYNOTE–799) reported that pembrolizumab plus concurrent chemoradiation therapy had good efficacy and safety for stage III NSCLC (79). The IMpower010 trial evaluated atezolizumab after chemotherapy in patients with resected stage IB–IIIA NSCLC. The results demonstrated that patients with stage II–IIIA NSCLC and PD–L1 TPS of at least 1% benefited from atezolizumab (80). Recently, the interim results of a phase 3 trial (GEMSTONE–301) reported that an anti–PD–L1 antibody (sugemalimab) after chemoradiotherapy prolonged PFS in patients with stage III NSCLC (81). For patients with advanced NSCLC who could not tolerate concurrent chemoradiotherapy, sequential chemoradiotherapy was a standard treatment. In August 2022, the phase 2 PACFIC–6 trial reported durvalumab after sequential chemoradiotherapy achieved acceptable safety and encouraging efficacy (82). The phase 3 PACFIC–5 trial is ongoing to further evaluate the efficacy and safety of durvalumab after sequential or concurrent chemoradiotherapy for unresectable stage III NSCLC (82).

Clinical trials have provided preliminary evidence supporting the efficacy and safety of anti–PD–(L)1 therapy plus/after radical treatments. Additional studies are needed to determine the optimal dosing strategy and duration of immunotherapy and to clarify the subgroup of patients who would benefit from adjuvant immunotherapy.

### Neoadjuvant anti–PD–(L)1 therapy

In recent years, several trials have evaluated anti–PD–(L)1 monotherapy or combined with chemotherapy or SABR as neoadjuvant treatment for resectable NSCLC. In 2018, a pilot study reported that neoadjuvant nivolumab therapy resulted in a major pathological response (MPR) of 45% and the response was correlated with TMB (83). Recently, neoadjuvant pembrolizumab therapy for resectable NSCLC has also been reported to be effective (84, 85). Furthermore, combined neoadjuvant therapy including ICI further improved efficacy. In 2020, a phase 2 trial reported that atezolizumab plus carboplatin and nab–paclitaxel as neoadjuvant therapy achieved a MPR rate of 57% for patients with stage IB to IIIA NSCLC (86). The phase 2 NADIM trial evaluated neoadjuvant nivolumab plus chemotherapy for stage IIIA NSCLC and reported a 2–year PFS of 77.1% (87). The SAKK 16/14 phase 2 trial reported that the addition of perioperative durvalumab to neoadjuvant chemotherapy yielded a MPR of 62% and a 1–year event–free survival (EFS) rate of 73% (88). A phase 3 trial (AEGEAN) is ongoing to further evaluate neoadjuvant durvalumab plus chemotherapy followed by adjuvant durvalumab (89). Another phase 2 trial reported that neoadjuvant toripalimab (an anti–PD–1 antibody) combined with chemotherapy achieved similar results (90). The first phase 3 trial evaluating neoadjuvant anti–PD–(L)1 therapy was CheckMate 816, which published the results in May 2022. This trial demonstrated that neoadjuvant nivolumab plus chemotherapy produced a median EFS of 31.6 months and a complete pathological response rate of 24% for patients with stage IB to IIIA resectable NSCLC (91). Recently, neoadjuvant therapy including both ICI and radiotherapy has been a research hotspot. A phase 2 trial reported that the addition of SABR (24 Gy in three fractions) to neoadjuvant durvalumab therapy resulted in much higher MPR (53.3% vs. 6.7%) in patients with stage I–IIIA NSCLC (92). Furthermore, the ongoing SQUAT phase trial is evaluating durvalumab plus CCRT (50 Gy in 25 fractions) as neoadjuvant therapy for patients with stage IIIA to IIIB resectable NSCLC (93).

In recent years, neoadjuvant immunotherapy has been investigated more than adjuvant immunotherapy. This may be due to 1) the existing tumor causing an immune response; 2) immunotherapy synergies with chemotherapy and/or radiotherapy; 3) the preoperative immunity may be stronger than postoperative immunity. Therefore, neoadjuvant immunotherapy is theoretically superior to adjuvant immunotherapy. Combined neoadjuvant therapy achieves high MPR, suggesting that combined therapy might be an optimal option for most patients. However, more clinical evidence is still needed to determine the indications and combination strategy of neoadjuvant immunotherapy for patients with resectable NSCLC.

### Anti–PD–(L)1 for SCLC

SCLC is highly aggressive and easily develops resistance to antitumor therapies. The standard systemic treatment for SCLC was etoposide plus platinum chemotherapy for several years. The high TMB of SCLC leads to a high neoantigen load, thus promoting potential antitumor immunity (94). In 2016, a phase 3 trial evaluated 1^st^–line ipilimumab plus chemotherapy for extensive–stage SCLC (ES–SCLC), but the results were negative (95). With evidence of the promising antitumor activity of anti–PD–(L)1 therapy, novel treatment strategies have been evaluated to improve the prognosis of patients with SCLC. Furthermore, because SCLC is heterogeneous and can be classified into four molecular subtypes, future studies are acquired to establish optimal management of SCLC in different subtypes (96).

### Anti–PD–(L)1 for previously treated extensive–stage SCLC

SCLC patients who advance after 1^st^–line chemotherapy have limited treatment options. In 2017, the KEYNOTE–028 phase 1 trial reported pembrolizumab had promising efficacy in patients with previously treated, PD–L1–expressing SCLC (97). Furthermore, a pooled analysis of the data from KEYNOTE–028 and KEYNOTE–158 suggested that the antitumor activity of pembrolizumab was independent of PD–L1 expression (98). The efficacy and safety of nivolumab was also demonstrated in the CheckMate 032 trial (99). Anti–PD–(L)1 therapy plus antiangiogenic therapy or chemotherapy alone could further improve prognosis. The phase 2 PASSION trial demonstrated that camrelizumab (an anti–PD–1 antibody) plus apatinib was effective and safe for previously treated patients with SCLC (100). A phase 2 trial reported that pembrolizumab plus paclitaxel showed moderate activity in previously treated patients with SCLC (101). A phase 1/2 trial evaluated rovalpituzumab tesirine (an antibody–drug conjugate) plus nivolumab with/without ipilimumab for previously treated patients with ES–SCLC. However, the toxicity was not tolerable (102).

Some trials were conducted to evaluate dual–ICIs for previously treated patients with SCLC. In 2016, the CheckMate 032 phase 1/2 trial reported that nivolumab monotherapy and nivolumab plus ipilimumab had antitumor activity and manageable toxicity for SCLC patients previously treated (103). However, the following CheckMate 451 phase 3 trial demonstrated that dual–ICIs were not superior to nivolumab monotherapy as maintenance therapy after first–line chemotherapy for ES–SCLC (104). The results and basic research suggested that patients with high TMB could benefit from dual–ICIs (104, 105). A phase 2 trial reported that the addition of SABR to durvalumab and tremelimumab did not improve the prognosis of patients with recurrent SCLC (106). Furthermore, a phase 1 trial suggested that quavonlimab plus pembrolizumab showed antitumor activity in previously treated patients with ES–SCLC (107).

The 2^nd^–line chemotherapy frequently used for SCLC included topotecan and amrubicin. The IFCT–1603 and CheckMate 331 respectively compared atezolizumab and nivolumab with chemotherapy as 2^nd^–line therapy for patients with SCLC. However, the results showed that anti–PD–(L)1 therapy was not superior to chemotherapy (108, 109).

Anti–PD–(L)1 monotherapy showed antitumor activity for previously treated SCLC. However, the efficacy of 2^nd^–line anti–PD–(L)1 therapy was not superior to chemotherapy. Dual–ICIs did not achieve better efficacy than monotherapy. Antiangiogenic therapy, chemotherapy, or radiation therapy may improve the prognosis, but more clinical evidence is warranted. Furthermore, some trials are ongoing to evaluate anti–PD–(L)1 combined with novel drugs (e.g., LAG–3 blockades and T cell immunoglobulin and mucin domain 3 blockades) for ES–SCLC (94).

### First–line anti–PD–(L)1 for extensive–stage SCLC

Potential antitumor immunity of previously untreated patients with SCLC may be more potent than that of heavily treated patients. Therefore, some trials evaluated 1^st^–line anti–PD–(L)1 therapy plus standard chemotherapy for patients with ES–SCLC. In 2018, the phase 3 IMpower133 trial demonstrated that the addition of atezolizumab improved OS and PFS (20). The subgroup analysis suggested that the efficacy was regardless of the expression of PD–L1 or TMB (110). The CASPIAN trial demonstrated that 1^st^–line durvalumab plus chemotherapy yielded better OS than chemotherapy alone (111). However, dual–ICIs (durvalumab and tremelimumab) plus chemotherapy did not improve prognosis than durvalumab plus chemotherapy (112). The KEYNOTE–604 trial demonstrated that the addition of pembrolizumab to chemotherapy also improved PFS (113). Similarly, the phase 3 CAPSTONE–1 trial reported that the addition of adebrelimab (a new anti–PD–L1 antibody) to chemotherapy improved OS (114). A phase 1 trial reported that pembrolizumab plus thoracic radiotherapy after chemotherapy was tolerated for SCLC, but its efficacy was unclear (115).

Anti–PD–(L)1 plus chemotherapy has been shown to be an effective and safe 1^st^–line treatment for ES–SCLC. Currently, further studies are needed to determine the optimal dosing strategy, duration of treatment, and patient selection. Because thoracic radiotherapy after chemotherapy improves the prognosis of ES–SCLC, the combination of anti–PD–(L)1 plus radiotherapy after chemotherapy should be further evaluated (116). Combination therapy including other treatments (e.g., antiangiogenic therapy and anti–TIGIT–antibodies) may further improve the prognosis, but clinical evidence is lacking.

### Anti–PD–(L)1 for limited–stage SCLC

The standard treatment for limited–stage SCLC (LS–SCLC) used to be CCRT. In recent years, several trials have evaluated ICIs plus or after CCRT for LS–SCLC. In 2020, a phase 1/2 trial reported that pembrolizumab plus CCRT resulted in favorable efficacy and safety (117). The STIMULI trial evaluated dual–ICIs after CCRT for LS–SCLC but reported negative results (118). Currently, results from phase 3 trials are lacking, trials including ADRIATIC and AdvanTIG 204 are currently evaluating anti–PD–(L)1, dual–ICIs, and ICI combined with anti–TIGIT antibody plus CCRT for LS–SCLC (119).

### Other immunotherapies

In addition to ICIs, lung cancer immunotherapies included vaccination and ACT. Vaccination was generally used for maintenance treatment to enhance the effect of chemotherapy or salvage therapy. In recent years, the combination of vaccine and ICI has been evaluated for lung cancer. The clinical value of some therapeutic vaccines (e.g., TG 4010, BLP25, NEO–PV–01) has been demonstrated (120–124). Vaccines can promote antitumor activity through multiple pathways, so they may synergize with other therapies. More basic and clinical studies are needed to explore the mechanisms of tumor immunology and develop predictive models to develop individual treatments for different subgroups of patients.

ACT has shown anti–tumor activity as salvage therapy for some patients with lung cancer. In recent years, TIL–based ACT has been evaluated for lung cancer. A phase 1 trial reported that TIL therapy was safe and showed a deep and durable response in some patients (125). Furthermore, chimeric antigen receptor (CAR)–modified ACT also showed antitumor immunity in some studies (126, 127). ACT based on novel technique was an effective salvage therapy for some patients with lung cancer. However, the response rate is not satisfactory and clinical evidence is lacking. Additional studies are needed to determine the patients suitable for ACT and to provide more evidence.

### Journals, countries, institutions, and authors

*Lung Cancer* was the most productive journal on lung cancer immunotherapy. Among the top 10 productive journals, *Lung Cancer* had the second highest citation indexes, and the *Journal of Thoracic Oncology* had the highest citation indexes. These two journals were both productive and impactful. The *N Engl J Med* was the most impactful journal in this area. The results of the correlation test suggested papers published in most of the journals with high 5–year IF or JCI are more likely to be highly cited. Among the 27 journals with the top papers, 22 were considered major journals. The articles published in major journals were likely to be impactful. Notably, most of the top papers were published in comprehensive journals, which may be due to the high IFs of these journals.

Researchers from China contributed most of the studies. However, papers by corresponding authors from the USA were much more influential. International collaboration was rare in China and Japan. In contrast, most of the top papers were contributed by authors from multiple countries/regions. Although some studies from developing countries have been published in recent years, studies from Africa or the Middle East are lacking. The most productive institution was the University of Texas MD Anderson Cancer Center. Although some universities in China contributed to many articles, their TPRs were low. Some institutions in developed countries, although their total number of papers was not high, contributed to many top papers. The most productive authors of the top papers were Reck M, Hellmann MD, and Horn L.

This study described the most influential journals, countries, institutions, and authors on lung cancer immunotherapy and presented collaboration networks. The results can help researchers select target journals for publication and find potential cooperative partners.

### Research trends and hotspots

Based on thousands of publications, this bibliometric analysis quantitatively and comprehensively presented research trends, status, and hotspots in lung cancer immunotherapy. Other major review methods, such as systematic literature review and meta–analysis, are unapplicable for this purpose (4).

This study analyzed the research trends. Before 2015, most publications on lung cancer immunotherapy focused on vaccination. The number of publications on vaccination or ACT varied little from year to year. The number of articles on ipilimumab has gradually increased since 2013. From 2015, anti–PD–1 antibodies became the main focus of research, followed by anti–PD–L1 antibodies. In recent years, additional studies have evaluated 1^st^–line immunotherapy or combined therapy compared to 2^nd^–line immunotherapy or monotherapy. Adjuvant and neoadjuvant therapy have recently become hotspots. Radiotherapy combined with ICI has recently been a research hotspot, and durvalumab plus radiotherapy was evaluated most compared with other ICIs.

The current research status on lung cancer immunotherapy is: 1) Anti–PD–(L)1 with/without chemotherapy is a standard 1^st^–line treatment for advanced NSCLC; 2) 1^st^– or 2^nd^–line anti–PD–(L)1 plus new immunotherapies may overcome resistance, but high–quality clinical evidence is lacking; 3) neoadjuvant and adjuvant anti–PD–(L)1 therapy has proven to be beneficial, and combined neoadjuvant anti–PD–(L)1 achieves encouraging efficacy; 4) anti–PD–(L)1 plus standard chemotherapy as first– or second–line treatment has favorable efficacy for ES–SCLC; 5) ICI plus CCRT may improve the efficacy for LS–SCLC, but more clinical evidence is needed; and 6) other immunotherapies are effective supplements to anti–PD–(L)1 therapy, and selected patients may benefit from them.

Current research hotspots include: 1) treatment for special patients; 2) treatment for patients who failed after anti–PD–(L)1 therapy; 3) immunotherapy combined with antiangiogenic therapy or radiotherapy; 4) combined neoadjuvant therapy; 5) anti–PD–(L)1 plus CCRT; 6) tumor immune microenvironment and immune–resistance mechanisms; and 7) new immunotherapies. The authors suggest that important future research directions include: 1) the optimal and individual managements for advanced NSCLC and ES–SCLC; 2) basic research and novel treatments to overcome resistance; 3) phase 3 clinical trials evaluating immunotherapy for LS–SCLC or resectable NSCLC; 4) robust predictive models; and 5) mechanisms and efficacy of immunotherapy combined with other therapies.

### Limitations

This study has some limitations. 1) This study aims to present the landscape of clinical immunotherapy for lung cancer, and only includes papers directly related to this topic published between 2010 and 2022. Therefore, earlier papers were excluded. Although some basic or clinical publications may have contained the keywords in their abstracts, they were not directly related to the topic. The search strategy excluded them to ensure that the identified papers were directly related to the topic, thereby avoiding interference, and better presenting the real research landscape. 2) ​Some papers evaluating multiple cancers were excluded, which introduces a bias in this study. However, with a reasonable search strategy, it was impossible to include them while excluding some other irrelevant papers. 3) The citation number was influenced by various confounding factors (e.g., publication time, research area, journal, and author). Therefore, citation number could not accurately represent the influence of a paper. Most of the top papers in this study were published prior to 2020; hence, some recent important papers were omitted, and the top papers could not represent the latest research hotspots. To minimize the impact of publication time, the authors also analyzed the average citation per year of the papers. 4) Due to the large number of papers, it was impossible to read every publication and thoroughly analyze the subareas. Furthermore, the recently published important papers represented the latest research hotspots but were difficult to quantificationally identify. To better present the trends and status of the sub–areas, the authors evaluated the development of subareas and presented the most recent advances including reports from the American Society of Clinical Oncology Annual Meeting and the WCLC. 5) This study focused on clinical studies. Therefore, important basic research studies may have been omitted, and basic immunology was not discussed. 6) Finally, the literature search was conducted only based on the Web of Science database, and papers not included in this database were omitted. This may have led to selection bias and analytical errors.

## Conclusions

To our knowledge, this study is the first comprehensive and quantitative bibliometric analysis of original articles on lung cancer immunotherapy. This study demonstrates the research trends and hotspots based on the analysis of 2,941 publications and 100 top papers. In addition, researchers can benefit from the results for selecting target journals for publication of findings and establishing cooperative relationships. The authors suggest that important research directions include: 1) optimal and individual treatment for advanced NSCLC and ES–SCLC; 2) overcoming immune–resistance; 3) clinical trials for resectable NSCLC or LS–SCLC; 4) robust predictive models; and 5) immunotherapy combined with other therapies. This study can help researchers gain a comprehensive picture of the research landscape, historical development, and recent hotspots in lung cancer immunotherapy and can provide inspiration for future research.

## Data availability statement

The raw data supporting the conclusions of this article will be made available by the authors, without undue reservation.

## Author contributions

XZ and YanL contributed to the study conception. YanL analyzed the data. YanL, XuC, XH, XiC, SJ, YarL, ZZ, LL, BQ, and YC contributed to the literature review. YanL wrote the manuscript. All authors contributed to the article and approved the submitted version.

## Acknowledgement

The author Yanhao Liu would like to thank his wife, Mrs. Yujie Zhang, and cat, Mr. Milk Cap, for their constant companionship and support.

## Conflict of interest

The authors declare that the research was conducted in the absence of any commercial or financial relationships that could be construed as a potential conflict of interest.

## Publisher’s note

All claims expressed in this article are solely those of the authors and do not necessarily represent those of their affiliated organizations, or those of the publisher, the editors and the reviewers. Any product that may be evaluated in this article, or claim that may be made by its manufacturer, is not guaranteed or endorsed by the publisher.

## Supplementary material

Supplementary Figure 1 Historical direct citation network among the publications.

Supplementary Figure 2 Top 25 references with the strongest citation bursts on lung cancer immunotherapy.

Supplementary Figure 3 Citation relationship network of the publications with the strongest link strength.

Supplementary Figure 4 The trends of the keywords rank of lung cancer immunotherapy.

Supplementary Figure 5 Top 25 keywords with the strongest citation bursts on lung cancer immunotherapy.
