# Supplementary figures and images for: Global research landscape and trends of lung cancer immunotherapy: A bibliometric analysis
Source: Front Immunol. 2022 Dec 1;13:1032747. doi: 10.3389/fimmu.2022.1032747 (PMC9751816; doi:10.3389/fimmu.2022.1032747)

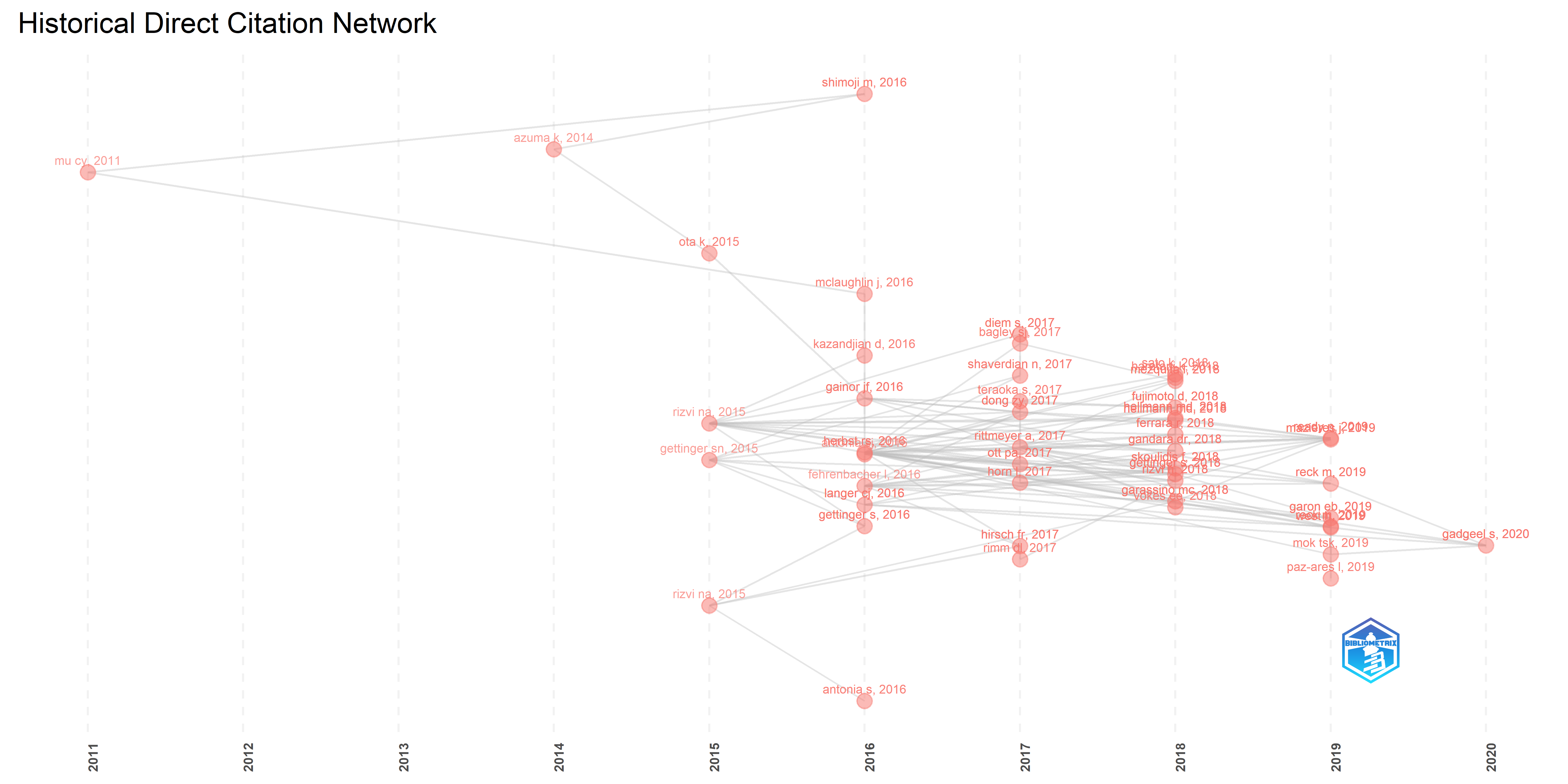

Supplement: Supplementary Figure 1 — Historical direct citation network among the publications. [file Image_1.png]

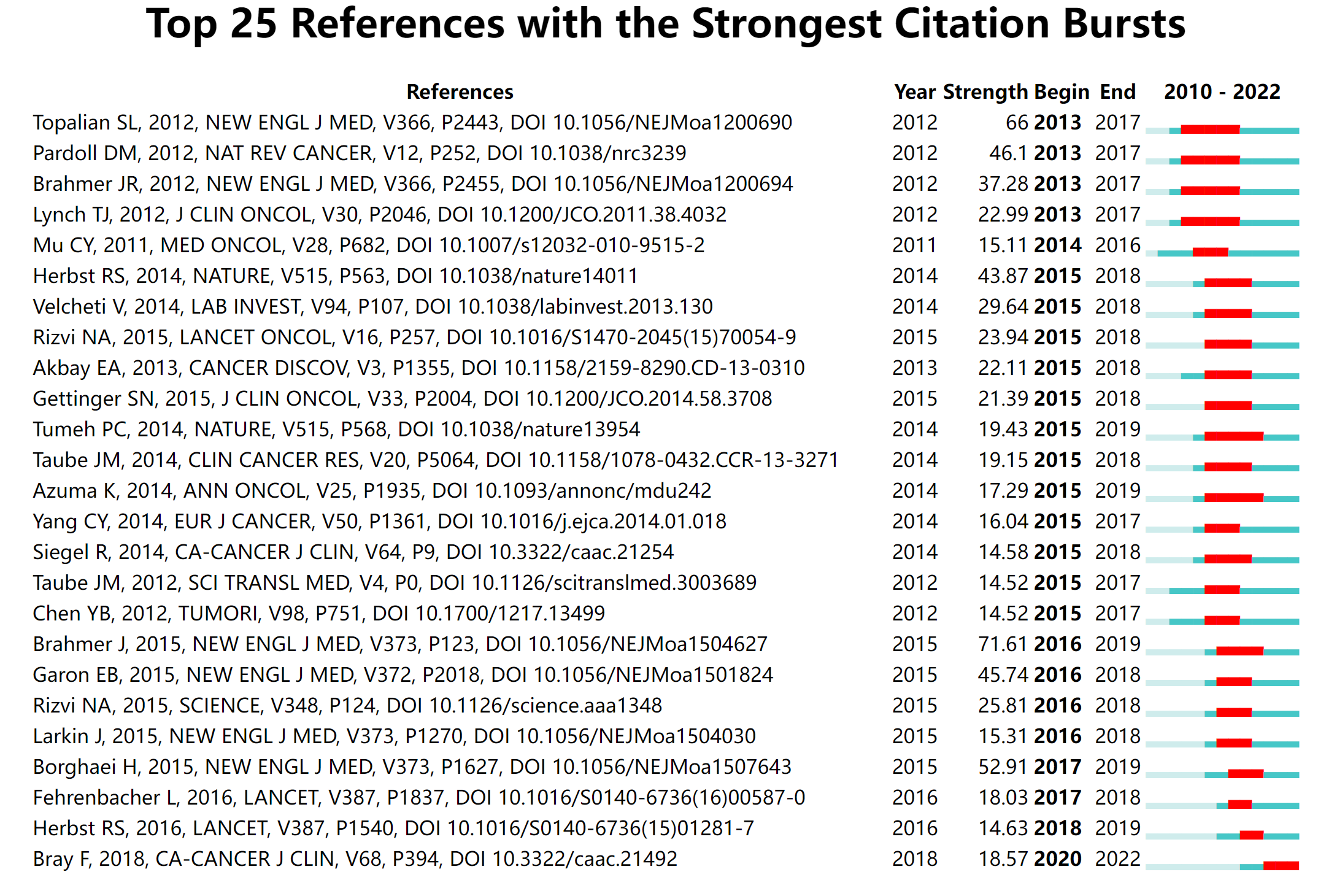

Supplement: Supplementary Figure 2 — Top 25 references with the strongest citation bursts on lung cancer immunotherapy. [file Image_2.png]

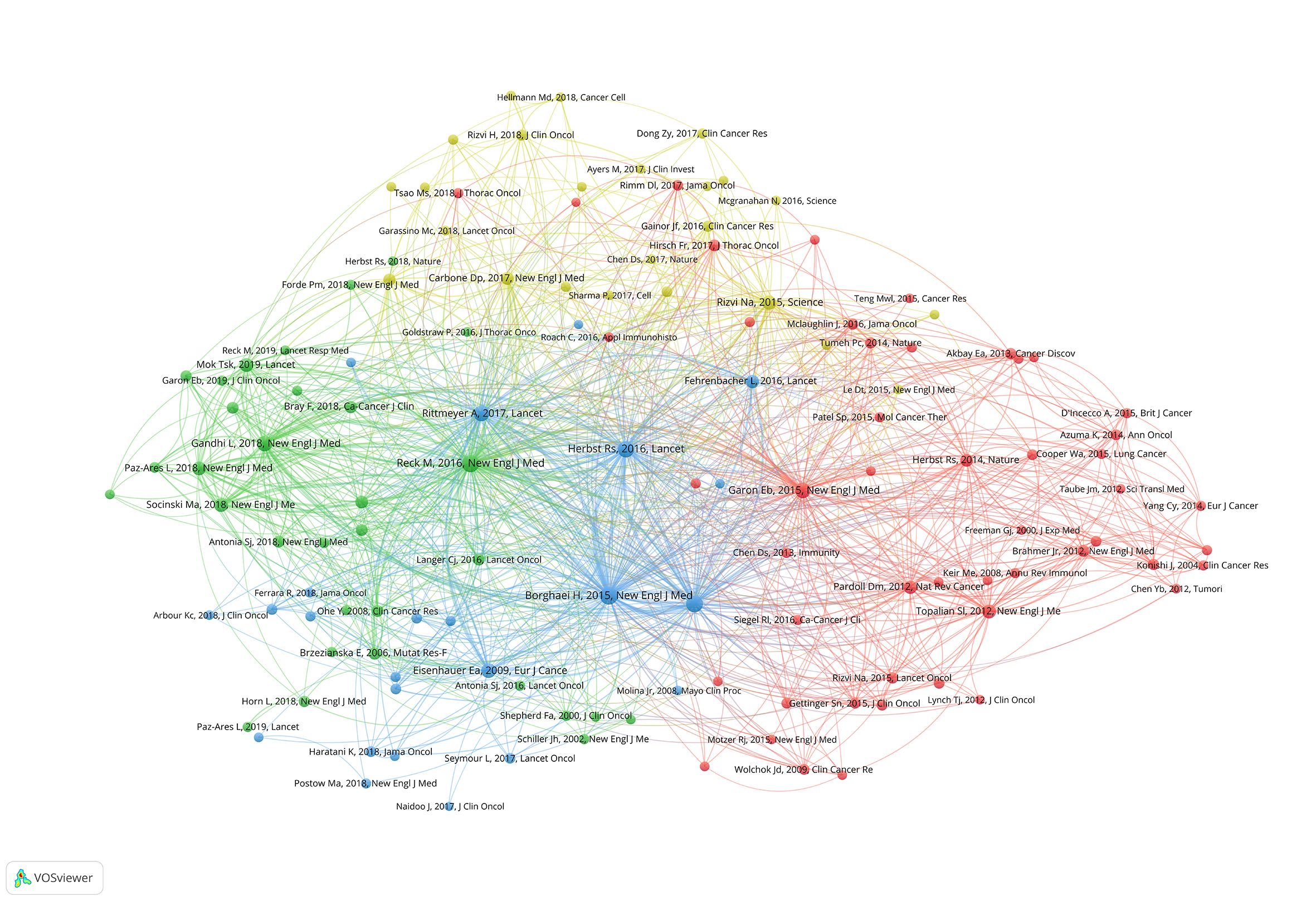

Supplement: Supplementary Figure 3 — Citation relationship network of the publications with the strongest link strength. [file Image_3.png]
