# Supplementary material for: Global research landscape and trends of lung cancer immunotherapy: A bibliometric analysis
Source: Front Immunol. 2022 Dec 1;13:1032747. doi: 10.3389/fimmu.2022.1032747 (PMC9751816; doi:10.3389/fimmu.2022.1032747)
Supplement: Supplementary Figure 4 — The trends of the keywords rank of lung cancer immunotherapy. [file DataSheet_1.docx]

**Search Strategy**

TI=(((lung) OR (pulmonary)) NEAR/0 ((cancer) OR (carcinoma) OR (neoplasm) OR (adenocarcinoma))) AND TI=((immunotherapy) OR (immunotherapies) OR (checkpoint inhibitor) OR (checkpoint inhibitors) OR (checkpoint blockade) OR (anti-programmed death 1 antibody) OR (PD1) OR (PD-1) OR (PD-L1) OR (anti-PD1) OR (anti-PD-1) OR (anti-PD-L1) OR (PD1/PD-L1) OR (PD-1/PD-L1) OR (anti-PD1/PD-L1) OR (anti-PD-1/PD-L1) OR (PD-(L)1) OR (BMS-936558) OR (ONO-4538) OR (atezolizumab) OR (avelumab) OR (durvalumab) OR (nivolumab) OR (pembrolizumab) OR (tislelizumab) OR (camrelizumab) OR (penpulimab) OR (toripalimab) OR (sintilimab) OR (lambrolizumab) OR (pidilizumab) OR (cemiplimab) OR (sugemalimab) OR (CTL-associated antigen-4) OR (cytotoxic T lymphocyte associated antigen 4) OR (CTLA-4) OR (ipilimumab) OR (tremelimumab) OR (anti-TIGIT antibody) OR (vibostolimab) OR (ociperlimab) OR (tiragolumab) OR (T-Cell NEAR/0 Transfer) OR (adoptive NEAR/0 cell NEAR/0 therapy) OR (CAR-T) OR (chimeric NEAR/0 antigen receptor T-cell NEAR/0 immunotherapy) OR (vaccine) OR (vaccination) OR (belagenpumatucel) OR (IDM-2101) OR (CIMAvax-EGF) OR (INGN-225) OR (TG4010) OR (ALT-803) OR (Tecemotide) OR (BLP25)) AND DT=(Article) NOT DT=(Book Chapter) NOT TI=((guideline) OR (consensus recommendations) OR (meta-analyses) OR (meta-analysis) OR (meta analysis) OR (data pooling) OR (pooled) OR (overview) OR (current status) OR (development) OR (review) OR (progress to date) OR (interstitial lung disease)) NOT TI=(lung NEAR/0 ((metastases OR metastasis)))
