# Supplementary material for: Global research landscape and trends of lung cancer immunotherapy: A bibliometric analysis
Source: Front Immunol. 2022 Dec 1;13:1032747. doi: 10.3389/fimmu.2022.1032747 (PMC9751816; doi:10.3389/fimmu.2022.1032747)
Supplement: Supplementary file 5 [file Table_1.docx]

|  | **TABLE S1** The 100 most cited papers in lung cancer immunotherapy. | | | | | | | |
| --- | --- | --- | --- | --- | --- | --- | --- | --- |
| Rank | | Title | Corresponding author | Journal | Year | Total citation | Average citation per year (rank) |  |
| 1 | | Nivolumab versus Docetaxel in Advanced Nonsquamous Non-Small-Cell Lung Cancer | Borghaei H | N. Engl. J. Med. | 2015 | 5854 | 878.1 (2) |  |
| 2 | | Pembrolizumab versus Chemotherapy for PD-L1-Positive Non-Small-Cell Lung Cancer | Brahmer JR | N. Engl. J. Med. | 2016 | 5287 | 946.9 (1) |  |
| 3 | | Mutational landscape determines sensitivity to PD-1 blockade in non-small cell lung cancer | Chan TA | Science | 2015 | 4908 | 684.8 (4) |  |
| 4 | | Nivolumab versus Docetaxel in Advanced Squamous-Cell Non-Small-Cell Lung Cancer | Brahmer J | N. Engl. J. Med. | 2015 | 4612 | 666.8 (5) |  |
| 5 | | Pembrolizumab for the Treatment of Non-Small-Cell Lung Cancer | Garon EB | N. Engl. J. Med. | 2015 | 3833 | 541.1 (6) |  |
| 6 | | Pembrolizumab versus docetaxel for previously treated, PD-L1-positive, advanced non-small-cell lung cancer (KEYNOTE-010): a randomised controlled trial | Herbst RS | Lancet | 2016 | 3217 | 521.7 (7) |  |
| 7 | | Pembrolizumab plus Chemotherapy in Metastatic Non-Small-Cell Lung Cancer | Gandhi L | N. Engl. J. Med. | 2018 | 2827 | 692.3 (3) |  |
| 8 | | Atezolizumab versus docetaxel in patients with previously treated non-small-cell lung cancer (OAK): a phase 3, open-label, multicentre randomised controlled trial | Gandara DR | Lancet | 2017 | 2647 | 488.7 (8) |  |
| 9 | | Durvalumab after Chemoradiotherapy in Stage III Non-Small-Cell Lung Cancer | Antonia SJ | N. Engl. J. Med. | 2017 | 2002 | 436.8 (10) |  |
| 10 | | Pembrolizumab plus Chemotherapy for Squamous Non-Small-Cell Lung Cancer | Paz-Ares L | N. Engl. J. Med. | 2018 | 1568 | 437.6 (9) |  |
| 11 | | Nivolumab plus Ipilimumab in Lung Cancer with a High Tumor Mutational Burden | Hellmann MD | N. Engl. J. Med. | 2018 | 1482 | 362.9 (12) |  |
| 12 | | First-Line Nivolumab in Stage IV or Recurrent Non-Small-Cell Lung Cancer | Carbone DP | N. Engl. J. Med. | 2017 | 1468 | 293.6 (15) |  |
| 13 | | Atezolizumab versus docetaxel for patients with previously treated non-small-cell lung cancer (POPLAR): a multicentre, open-label, phase 2 randomised controlled trial | Fehrenbacher L | Lancet | 2016 | 1454 | 235.8 (16) |  |
| 14 | | Pembrolizumab versus chemotherapy for previously untreated, PD-L1-expressing, locally advanced or metastatic non-small-cell lung cancer (KEYNOTE-042): a randomised, open-label, controlled, phase 3 trial | Mok TSK | Lancet | 2019 | 1260 | 408.6 (11) |  |
| 15 | | First-Line Atezolizumab plus Chemotherapy in Extensive-Stage Small-Cell Lung Cancer | Horn L | N. Engl. J. Med. | 2018 | 1174 | 335.4 (14) |  |
| 16 | | Activity and safety of nivolumab, an anti-PD-1 immune checkpoint inhibitor, for patients with advanced, refractory squamous non-small-cell lung cancer (CheckMate 063): a phase 2, single-arm trial | Rizvi NA | Lancet Oncol. | 2015 | 1030 | 142.1 (28) |  |
| 17 | | Carboplatin and pemetrexed with or without pembrolizumab for advanced, non-squamous non-small-cell lung cancer: a randomised, phase 2 cohort of the open-label KEYNOTE-021 study | Langer CJ | Lancet Oncol. | 2016 | 956 | 171.2 (21) |  |
| 18 | | Nivolumab plus Ipilimumab in Advanced Non-Small-Cell Lung Cancer | Hellmann MD | N. Engl. J. Med. | 2019 | 922 | 356.9 (13) |  |
| 19 | | Neoadjuvant PD-1 Blockade in Resectable Lung Cancer | Pardoll DM | N. Engl. J. Med. | 2018 | 809 | 198.1 (18) |  |
| 20 | | PD-L1 Immunohistochemistry Assays for Lung Cancer: Results from Phase 1 of the Blueprint PD-L1 IHC Assay Comparison Project | Hirsch FR | J. Thorac. Oncol. | 2017 | 809 | 151.7 (25) |  |
| 21 | | Nivolumab alone and nivolumab plus ipilimumab in recurrent small-cell lung cancer (CheckMate 032): a multicentre, open-label, phase 1/2 trial | Calvo E | Lancet Oncol. | 2016 | 785 | 132.7 (30) |  |
| 22 | | Ipilimumab in Combination With Paclitaxel and Carboplatin As First-Line Treatment in Stage IIIB/IV Non-Small-Cell Lung Cancer: Results From a Randomized, Double-Blind, Multicenter Phase II Study | Lynch TJ | J. Clin. Oncol. | 2012 | 746 | 74.6 (56) |  |
| 23 | | Molecular Determinants of Response to Anti-Programmed Cell Death (PD)-1 and Anti-Programmed Death-Ligand 1 (PD-L1) Blockade in Patients With Non-Small-Cell Lung Cancer Profiled With Targeted Next-Generation Sequencing | Hellmann MD | J. Clin. Oncol. | 2018 | 709 | 166.8 (22) |  |
| 24 | | EGFR Mutations and ALK Rearrangements Are Associated with Low Response Rates to PD-1 Pathway Blockade in Non-Small Cell Lung Cancer: A Retrospective Analysis | Gainor JF | Clin. Cancer Res. | 2016 | 660 | 114.8 (34) |  |
| 25 | | Pembrolizumab for patients with melanoma or non-small-cell lung cancer and untreated brain metastases: early analysis of a non-randomised, open-label, phase 2 trial | Goldberg SB | Lancet Oncol. | 2016 | 635 | 107.3 (40) |  |
| 26 | | Updated Analysis of KEYNOTE-024: Pembrolizumab Versus Platinum-Based Chemotherapy for Advanced Non-Small-Cell Lung Cancer With PD-L1 Tumor Proportion Score of 50% or Greater | Reck M | J. Clin. Oncol. | 2019 | 627 | 192.9 (19) |  |
| 27 | | STK11/LKB1 Mutations and PD-1 Inhibitor Resistance in KRAS-Mutant Lung Adenocarcinoma | Heymach JV | Cancer Discov. | 2018 | 625 | 159.6 (24) |  |
| 28 | | Nivolumab plus ipilimumab as first-line treatment for advanced non-small-cell lung cancer (CheckMate 012): results of an open-label, phase 1, multicohort study | Hellmann MD | Lancet Oncol. | 2017 | 601 | 111 (38) |  |
| 29 | | Overall Survival and Long-Term Safety of Nivolumab (Anti-Programmed Death 1 Antibody, BMS-936558, ONO-4538) in Patients With Previously Treated Advanced Non-Small-Cell Lung Cancer | Gettinger SN | J. Clin. Oncol. | 2015 | 583 | 83.3 (51) |  |
| 30 | | Blood-based tumor mutational burden as a predictor of clinical benefit in non-small-cell lung cancer patients treated with atezolizumab | Shames DS | Nat. Med. | 2018 | 563 | 150.1 (27) |  |
| 31 | | Quantitative Assessment of the Heterogeneity of PD-L1 Expression in Non-Small-Cell Lung Cancer | Rimm DL | JAMA Oncol. | 2016 | 561 | 87.4 (47) |  |
| 32 | | Durvalumab plus platinum-etoposide versus platinum-etoposide in first-line treatment of extensive-stage small-cell lung cancer (CASPIAN): a randomised, controlled, open-label, phase 3 trial | Paz-Ares L | Lancet | 2019 | 553 | 214.1 (17) |  |
| 33 | | Previous radiotherapy and the clinical activity and toxicity of pembrolizumab in the treatment of non-small-cell lung cancer: a secondary analysis of the KEYNOTE-001 phase 1 trial | Lee P | Lancet Oncol. | 2017 | 552 | 112.3 (37) |  |
| 34 | | Atezolizumab in combination with carboplatin plus nab-paclitaxel chemotherapy compared with chemotherapy alone as first-line treatment for metastatic non-squamous non-small-cell lung cancer (IMpower130): a multicentre, randomised, open-label, phase 3 trial | Cappuzzo F | Lancet Oncol. | 2019 | 536 | 183.8 (20) |  |
| 35 | | Genomic Features of Response to Combination Immunotherapy in Patients with Advanced Non-Small-Cell Lung Cancer | Hellmann MD | Cancer Cell | 2018 | 497 | 121.7 (32) |  |
| 36 | | A Prospective, Multi-institutional, Pathologist-Based Assessment of 4 Immunohistochemistry Assays for PD-L1 Expression in Non-Small Cell Lung Cancer | Rimm DL | JAMA Oncol. | 2017 | 485 | 100.3 (43) |  |
| 37 | | Association of Immune-Related Adverse Events With Nivolumab Efficacy in Non-Small Cell Lung Cancer | Hayashi H | JAMA Oncol. | 2018 | 484 | 113.9 (35) |  |
| 38 | | An Abscopal Response to Radiation and Ipilimumab in a Patient with Metastatic Non-Small Cell Lung Cancer | Formenti SC | Cancer Immunol. Res. | 2013 | 481 | 56.6 (69) |  |
| 39 | | Evolution of Neoantigen Landscape during Immune Checkpoint Blockade in Non-Small Cell Lung Cancer | Velculescu VE | Cancer Discov. | 2017 | 446 | 85 (49) |  |
| 40 | | High expression of PD-L1 in lung cancer may contribute to poor prognosis and tumor cells immune escape through suppressing tumor infiltrating dendritic cells maturation | Huang JA | Med. Oncol. | 2011 | 446 | 41.5 (87) |  |
| 41 | | Association of PD-L1 overexpression with activating EGFR mutations in surgically resected nonsmall- cell lung cancer | Okamoto I | Ann. Oncol. | 2014 | 443 | 57.8 (68) |  |
| 42 | | Tumor Mutational Burden and Efficacy of Nivolumab Monotherapy and in Combination with Ipilimumab in Small-Cell Lung Cancer | Hellmann MD | Cancer Cell | 2018 | 441 | 108 (39) |  |
| 43 | | Potential Predictive Value of TP53 and KRAS Mutation Status for Response to PD-1 Blockade Immunotherapy in Lung Adenocarcinoma | Wu YL | Clin. Cancer Res. | 2017 | 423 | 84.6 (50) |  |
| 44 | | Safety and antitumour activity of durvalumab plus tremelimumab in non-small-cell lung cancer: a multicentre, phase 1b study | Rizvi NA | Lancet Oncol. | 2016 | 421 | 67.4 (61) |  |
| 45 | | Ipilimumab in combination with paclitaxel and carboplatin as first-line therapy in extensive-disease-small-cell lung cancer: results from a randomized, double-blind, multicenter phase 2 trial | Reck M | Ann. Oncol. | 2013 | 417 | 44.3 (82) |  |
| 46 | | PD-1 and PD-L1 expression in molecularly selected non-small-cell lung cancer patients | Cappuzzo F | Br. J. Cancer | 2015 | 410 | 55.3 (71) |  |
| 47 | | Five-Year Overall Survival for Patients With Advanced Non-Small-Cell Lung Cancer Treated With Pembrolizumab: Results From the Phase I KEYNOTE-001 Study | Garon EB | J. Clin. Oncol. | 2019 | 404 | 151.5 (26) |  |
| 48 | | Control of PD-L1 Expression by Oncogenic Activation of the AKT-mTOR Pathway in Non-Small Cell Lung Cancer | Dennis PA | Cancer Res. | 2016 | 399 | 62.2 (66) |  |
| 49 | | Atezolizumab plus bevacizumab and chemotherapy in non-small-cell lung cancer (IMpower150): key subgroup analyses of patients with EGFR mutations or baseline liver metastases in a randomised, open-label phase 3 trial | Reck M | Lancet Resp. Med. | 2019 | 392 | 127.1 (31) |  |
| 50 | | Nivolumab Versus Docetaxel in Previously Treated Patients With Advanced Non-Small-Cell Lung Cancer: Two-Year Outcomes From Two Randomized, Open-Label, Phase III Trials (CheckMate 017 and CheckMate 057) | Horn L | J. Clin. Oncol. | 2017 | 387 | 86 (48) |  |
| 51 | | Hyperprogressive Disease in Patients With Advanced Non-Small Cell Lung Cancer Treated With PD-1/PD-L1 Inhibitors or With Single-Agent Chemotherapy | Besse B | JAMA Oncol. | 2018 | 380 | 106 (41) |  |
| 52 | | Five-Year Follow-Up of Nivolumab in Previously Treated Advanced Non-Small-Cell Lung Cancer: Results From the CA209-003 Study | Gettinger S | J. Clin. Oncol. | 2018 | 377 | 94.3 (44) |  |
| 53 | | Negative association of antibiotics on clinical activity of immune checkpoint inhibitors in patients with advanced renal cell and non-small-cell lung cancer | Routy B | Ann. Oncol. | 2018 | 373 | 93.3 (45) |  |
| 54 | | Proliferation of PD-1+CD8 T cells in peripheral blood after PD-1-targeted therapy in lung cancer patients | Ahmed R | Proc. Natl. Acad. Sci. U. S. A. | 2017 | 373 | 73.4 (58) |  |
| 55 | | Neutrophil-to-Lymphocyte ratio (NLR) and Platelet-to-Lymphocyte ratio (PLR) as prognostic markers in patients with non-small cell lung cancer (NSCLC) treated with nivolumab | Diem S | Lung Cancer | 2017 | 372 | 78.3 (54) |  |
| 56 | | Radiotherapy induces responses of lung cancer to CTLA-4 blockade | Formenti SC;Demaria S | Nat. Med. | 2018 | 357 | 102 (42) |  |
| 57 | | Association of the Lung Immune Prognostic Index With Immune Checkpoint Inhibitor Outcomes in Patients With Advanced Non-Small Cell Lung Cancer | Besse B | JAMA Oncol. | 2018 | 350 | 82.4 (52) |  |
| 58 | | Immune checkpoint inhibitors for patients with advanced lung cancer and oncogenic driver alterations: results from the IMMUNOTARGET registry | Mazieres J | Ann. Oncol. | 2019 | 341 | 120.4 (33) |  |
| 59 | | Tecemotide (L-BLP25) versus placebo after chemoradiotherapy for stage III non-small-cell lung cancer (START): a randomised, double-blind, phase 3 trial | Butts C | Lancet Oncol. | 2014 | 333 | 39.6 (89) |  |
| 60 | | Nivolumab Monotherapy for First-Line Treatment of Advanced Non-Small-Cell Lung Cancer | Gettinger S | J. Clin. Oncol. | 2016 | 322 | 56 (70) |  |
| 61 | | Antibody-mediated thyroid dysfunction during T-cell checkpoint blockade in patients with non-small-cell lung cancer | Hellmann MD | Ann. Oncol. | 2017 | 316 | 60.2 (67) |  |
| 62 | | Induction of PD-L1 Expression by the EML4-ALK Oncoprotein and Downstream Signaling Pathways in Non-Small Cell Lung Cancer | Okamoto I | Clin. Cancer Res. | 2015 | 315 | 46.7 (80) |  |
| 63 | | Effect of Pembrolizumab After Stereotactic Body Radiotherapy vs Pembrolizumab Alone on Tumor Response in Patients With Advanced Non-Small Cell Lung Cancer: Results of the PEMBRO-RT Phase 2 Randomized Clinical Trial | Theelen WSME | JAMA Oncol. | 2019 | 310 | 112.7 (36) |  |
| 64 | | Nivolumab in Combination With Platinum-Based Doublet Chemotherapy for First-Line Treatment of Advanced Non-Small-Cell Lung Cancer | Rizvi NA | J. Clin. Oncol. | 2016 | 305 | 53 (73) |  |
| 65 | | Durvalumab as third-line or later treatment for advanced non-small-cell lung cancer (ATLANTIC): an open-label, single-arm, phase 2 study | Garassino MC | Lancet Oncol. | 2018 | 300 | 72 (59) |  |
| 66 | | Impaired HLA Class I Antigen Processing and Presentation as a Mechanism of Acquired Resistance to Immune Checkpoint Inhibitors in Lung Cancer | Gettinger S | Cancer Discov. | 2017 | 289 | 64.2 (62) |  |
| 67 | | Pretreatment neutrophil-to-lymphocyte ratio as a marker of outcomes in nivolumab-treated patients with advanced non-small-cell lung cancer | Bagley SJ | Lung Cancer | 2017 | 285 | 55.2 (72) |  |
| 68 | | Pembrolizumab in Patients With Extensive-Stage Small-Cell Lung Cancer: Results From the Phase Ib KEYNOTE-028 Study | Ott PA | J. Clin. Oncol. | 2017 | 283 | 62.9 (64) |  |
| 69 | | Updated Analysis From KEYNOTE-189: Pembrolizumab or Placebo Plus Pemetrexed and Platinum for Previously Untreated Metastatic Nonsquamous Non-Small-Cell Lung Cancer | Gadgeel S | J. Clin. Oncol. | 2020 | 278 | 133.4 (29) |  |
| 70 | | Phase III Randomized Trial of Ipilimumab Plus Etoposide and Platinum Versus Placebo Plus Etoposide and Platinum in Extensive-Stage Small-Cell Lung Cancer | Reck M | J. Clin. Oncol. | 2016 | 272 | 48.7 (77) |  |
| 71 | | Nivolumab versus docetaxel in previously treated advanced non-small-cell lung cancer (CheckMate 017 and CheckMate 057): 3-year update and outcomes in patients with liver metastases | Vokes EE | Ann. Oncol. | 2018 | 262 | 62.9 (65) |  |
| 72 | | FDA Approval Summary: Nivolumab for the Treatment of Metastatic Non-Small Cell Lung Cancer With Progression On or After Platinum-Based Chemotherapy | Kazandjian D | Oncologist | 2016 | 261 | 42.9 (83) |  |
| 73 | | First-Line Nivolumab Plus Ipilimumab in Advanced Non-Small-Cell Lung Cancer (CheckMate 568): Outcomes by Programmed Death Ligand 1 and Tumor Mutational Burden as Biomarkers | Ready N | J. Clin. Oncol. | 2019 | 255 | 80.5 (53) |  |
| 74 | | Avelumab versus docetaxel in patients with platinum-treated advanced non-small-cell lung cancer (JAVELIN Lung 200): an open-label, randomised, phase 3 study | Park K | Lancet Oncol. | 2018 | 243 | 67.8 (60) |  |
| 75 | | Phase II Trial of Atezolizumab As First-Line or Subsequent Therapy for Patients With Programmed Death-Ligand 1-Selected Advanced Non-Small-Cell Lung Cancer (BIRCH) | Felip E | J. Clin. Oncol. | 2017 | 231 | 47.8 (78) |  |
| 76 | | Assessment of Blood Tumor Mutational Burden as a Potential Biomarker for Immunotherapy in Patients With Non-Small Cell Lung Cancer With Use of a Next-Generation Sequencing Cancer Gene Panel | Wang J | JAMA Oncol. | 2019 | 229 | 74.3 (57) |  |
| 77 | | Adjuvant MAGE-A3 Immunotherapy in Resected Non-Small-Cell Lung Cancer: Phase II Randomized Study Results | Vansteenkiste J | J. Clin. Oncol. | 2013 | 228 | 25.6 (97) |  |
| 78 | | Early Immune-Related Adverse Events and Association with Outcome in Advanced Non-Small Cell Lung Cancer Patients Treated with Nivolumab: A Prospective Cohort Study | Fujimoto D | J. Thorac. Oncol. | 2017 | 226 | 50.2 (74) |  |
| 79 | | Correlation between immune-related adverse events and efficacy in non-small cell lung cancer treated with nivolumab | Akamatsu H | Lung Cancer | 2018 | 220 | 49.8 (76) |  |
| 80 | | FDA Approval Summary: Pembrolizumab for the Treatment of Patients With Metastatic Non-Small Cell Lung Cancer Whose Tumors Express Programmed Death-Ligand 1 | Sul J | Oncologist | 2016 | 218 | 35.8 (91) |  |
| 81 | | First-line nivolumab plus ipilimumab combined with two cycles of chemotherapy in patients with non-small-cell lung cancer (CheckMate 9LA): an international, randomised, open-label, phase 3 trial | Paz-Ares L | Lancet Oncol. | 2021 | 215 | 161.3 (23) |  |
| 82 | | Phase III Trial of Ipilimumab Combined With Paclitaxel and Carboplatin in Advanced Squamous Non-Small-Cell Lung Cancer | Govindan R | J. Clin. Oncol. | 2017 | 211 | 45.2 (81) |  |
| 83 | | Avelumab for patients with previously treated metastatic or recurrent non-small-cell lung cancer (JAVELIN Solid Tumor): dose-expansion cohort of a multicentre, open-label, phase 1b trial | Gulley JL | Lancet Oncol. | 2017 | 211 | 41.5 (86) |  |
| 84 | | Antibody-Fc/FcR Interaction on Macrophages as a Mechanism for Hyperprogressive Disease in Non-small Cell Lung Cancer Subsequent to PD-1/PD-L1 Blockade | Sozzi G | Clin. Cancer Res. | 2019 | 210 | 63 (63) |  |
| 85 | | Five-Year Survival and Correlates Among Patients With Advanced Melanoma, Renal Cell Carcinoma, or Non-Small Cell Lung Cancer Treated With Nivolumab | Topalian SL | JAMA Oncol. | 2019 | 208 | 78 (55) |  |
| 86 | | Immune-Related Gene Expression Profiling After PD-1 Blockade in Non-Small Cell Lung Carcinoma, Head and Neck Squamous Cell Carcinoma, and Melanoma | Prat A | Cancer Res. | 2017 | 208 | 42.3 (84) |  |
| 87 | | CD47-blocking immunotherapies stimulate macrophage-mediated destruction of small-cell lung cancer | Weiskopf K;Weissman IL;Sage J | J. Clin. Invest. | 2016 | 201 | 34 (93) |  |
| 88 | | Therapeutic regulation of myeloid-derived suppressor cells and immune response to cancer vaccine in patients with extensive stage small cell lung cancer | Gabrilovich D | Cancer Immunol. Immunother. | 2013 | 201 | 22.1 (99) |  |
| 89 | | Changes in serum interleukin-8 (IL-8) levels reflect and predict response to anti-PD-1 treatment in melanoma and non-small-cell lung cancer patients | Melero I | Ann. Oncol. | 2017 | 194 | 40.1 (88) |  |
| 90 | | ALT-803, an IL-15 superagonist, in combination with nivolumab in patients with metastatic non-small cell lung cancer: a non-randomised, open-label, phase 1b trial | Rubinstein MP | Lancet Oncol. | 2018 | 192 | 47 (79) |  |
| 91 | | Durvalumab With or Without Tremelimumab vs Standard Chemotherapy in First-line Treatment of Metastatic Non-Small Cell Lung Cancer The MYSTIC Phase 3 Randomized Clinical Trial | Rizvi NA | JAMA Oncol. | 2020 | 190 | 91.2 (46) |  |
| 92 | | Therapeutic vaccination with TG4010 and first-line chemotherapy in advanced non-small-cell lung cancer: a controlled phase 2B trial | Quoix E | Lancet Oncol. | 2011 | 187 | 17.7 (100) |  |
| 93 | | EGFR mutation correlates with uninflamed phenotype and weak immunogenicity, causing impaired response to PD-1 blockade in non-small cell lung cancer | Zhong WZ;Wu YL | OncoImmunology | 2017 | 184 | 34 (94) |  |
| 94 | | Whole body PD-1 and PD-L1 positron emission tomography in patients with non-small-cell lung cancer | deLangen AJ | Nat. Commun. | 2018 | 179 | 50 (75) |  |
| 95 | | Early Assessment of Lung Cancer Immunotherapy Response via Circulating Tumor DNA | Patel AA | Clin. Cancer Res. | 2018 | 175 | 42 (85) |  |
| 96 | | The Clinical Relevance of the miR-197/CKS1B/STAT3-mediated PD-L1 Network in Chemoresistant Non-small-cell Lung Cancer | Ochiya T | Mol. Ther. | 2015 | 175 | 24.4 (98) |  |
| 97 | | Monitoring PD-L1 positive circulating tumor cells in non-small cell lung cancer patients treated with the PD-1 inhibitor Nivolumab | Gazzaniga P | Sci Rep | 2016 | 173 | 29.7 (95) |  |
| 98 | | Tumor immune microenvironment and nivolumab efficacy in EGFR mutation-positive non-small-cell lung cancer based on T790M status after disease progression during EGFR-TKI treatment | Hayashi H | Ann. Oncol. | 2017 | 172 | 35 (92) |  |
| 99 | | Comparison of the Toxicity Profile of PD-1 Versus PD-L1 Inhibitors in Non-Small Cell Lung Cancer: A Systematic Analysis of the Literature | Ramalingam SS | Cancer | 2018 | 170 | 38.5 (90) |  |
| 100 | | HDAC Inhibitors Enhance T-Cell Chemokine Expression and Augment Response to PD-1 Immunotherapy in Lung Adenocarcinoma | Beg AA | Clin. Cancer Res. | 2016 | 165 | 28.3 (96) |  |
