# Supplementary material for: Global research landscape and trends of lung cancer immunotherapy: A bibliometric analysis
Source: Front Immunol. 2022 Dec 1;13:1032747. doi: 10.3389/fimmu.2022.1032747 (PMC9751816; doi:10.3389/fimmu.2022.1032747)
Supplement: Supplementary file 7 [file Table_3.docx]

| **TABLE S3** \| The journals with top-cited papers in lung cancer immunotherapy from 2010 to 2022. | | | |
| --- | --- | --- | --- |
| **Journals with top-cited papers** | **Top-Cited Paper number** | **Paper number** | **TPR (2010-2020)** |
| J. Clin. Oncol. | 16 | 29 | 55.17% |
| Lancet Oncol. | 15 | 24 | 62.50% |
| N. Engl. J. Med. | 12 | 13 | 92.31% |
| JAMA Oncol. | 9 | 24 | 37.50% |
| Ann. Oncol. | 8 | 40 | 20.00% |
| Clin. Cancer Res. | 6 | 44 | 13.64% |
| Lancet | 5 | 7 | 71.43% |
| Cancer Discov. | 3 | 6 | 50.00% |
| Lung Cancer | 3 | 150 | 2.00% |
| Cancer Cell | 2 | 3 | 66.67% |
| Cancer Res. | 2 | 9 | 22.22% |
| J. Thorac. Oncol. | 2 | 60 | 3.33% |
| Nat. Med. | 2 | 6 | 33.33% |
| Oncologist | 2 | 24 | 8.33% |
| Br. J. Cancer | 1 | 9 | 11.11% |
| Cancer | 1 | 11 | 9.09% |
| Cancer Immunol. Immunother. | 1 | 80 | 1.25% |
| Cancer Immunol. Res. | 1 | 13 | 7.69% |
| J. Clin. Invest. | 1 | 2 | 50.00% |
| Lancet Resp. Med. | 1 | 5 | 20.00% |
| Med. Oncol. | 1 | 6 | 16.67% |
| Mol. Ther. | 1 | 2 | 50.00% |
| Nat. Commun. | 1 | 8 | 12.50% |
| OncoImmunology | 1 | 49 | 2.04% |
| Proc. Natl. Acad. Sci. U. S. A. | 1 | 1 | 100.00% |
| Science | 1 | 1 | 100.00% |
| Sci Rep | 1 | 39 | 2.56% |
