# Supplementary material for: Global research landscape and trends of lung cancer immunotherapy: A bibliometric analysis
Source: Front Immunol. 2022 Dec 1;13:1032747. doi: 10.3389/fimmu.2022.1032747 (PMC9751816; doi:10.3389/fimmu.2022.1032747)
Supplement: Supplementary file 8 [file Table_4.docx]

|  | **TABLE S4** The 110 papers in lung cancer immunotherapy published in top-journals since 2020. | | | | | |
| --- | --- | --- | --- | --- | --- | --- |
| No. | | Title | Corresponding author | Journal | Year | Total citation |
| 1 | | Updated Analysis From KEYNOTE-189: Pembrolizumab or Placebo Plus Pemetrexed and Platinum for Previously Untreated Metastatic Nonsquamous Non-Small-Cell Lung Cancer | Gadgeel S | J. Clin. Oncol. | 2020 | 282 |
| 2 | | First-line nivolumab plus ipilimumab combined with two cycles of chemotherapy in patients with non-small-cell lung cancer (CheckMate 9LA): an international, randomised, open-label, phase 3 trial | Paz-Ares L | Lancet Oncol. | 2021 | 215 |
| 3 | | Durvalumab With or Without Tremelimumab vs Standard Chemotherapy in First-line Treatment of Metastatic Non-Small Cell Lung Cancer The MYSTIC Phase 3 Randomized Clinical Trial | Rizvi NA | JAMA Oncol. | 2020 | 192 |
| 4 | | Neoadjuvant atezolizumab and chemotherapy in patients with resectable non-small-cell lung cancer: an open-label, multicentre, single-arm, phase 2 trial | Rizvi NA | Lancet Oncol. | 2020 | 159 |
| 5 | | Neoadjuvant chemotherapy and nivolumab in resectable non-small-cell lung cancer (NADIM): an open-label, multicentre, single-arm, phase 2 trial | Provencio M | Lancet Oncol. | 2020 | 143 |
| 6 | | TATTON: a multi-arm, phase lb trial of osimertinib combined with selumetinib, savolitinib, or durvalumab in EGFR-mutant lung cancer | Oxnard GR | Ann. Oncol. | 2020 | 138 |
| 7 | | Impact of PD-1 Blockade on Severity of COVID-19 in Patients with Lung Cancers | Hellmann MD | Cancer Discov. | 2020 | 134 |
| 8 | | Pembrolizumab or Placebo Plus Etoposide and Platinum as First-Line Therapy for Extensive-Stage Small-Cell Lung Cancer: Randomized, Double-Blind, Phase III KEYNOTE-604 Study | Rudin CM | J. Clin. Oncol. | 2020 | 134 |
| 9 | | Cemiplimab monotherapy for first-line treatment of advanced non-small-cell lung cancer with PD-L1 of at least 50%: a multicentre, open-label, global, phase 3, randomised, controlled trial | Sezer A | Lancet | 2021 | 105 |
| 10 | | Neoadjuvant nivolumab or nivolumab plus ipilimumab in operable non-small cell lung cancer: the phase 2 randomized NEOSTAR trial | Cascone T | Nat. Med. | 2021 | 92 |
| 11 | | Association Between Body Mass Index and Overall Survival With Immune Checkpoint Inhibitor Therapy for Advanced Non-Small Cell Lung Cancer | Kichenadasse G | JAMA Oncol. | 2020 | 92 |
| 12 | | Clinical and molecular correlates of PD-L1 expression in patients with lung adenocarcinomas | Hellmann MD | Ann. Oncol. | 2020 | 86 |
| 13 | | Five-Year Outcomes With Pembrolizumab Versus Chemotherapy for Metastatic Non-Small-Cell Lung Cancer With PD-L1 Tumor Proportion Score >= 50% | Reck M | J. Clin. Oncol. | 2021 | 85 |
| 14 | | Durvalumab, with or without tremelimumab, plus platinum-etoposide versus platinum-etoposide alone in first-line treatment of extensive-stage small-cell lung cancer (CASPIAN): updated results from a randomised, controlled, open-label, phase 3 trial | Paz-Ares L | Lancet Oncol. | 2021 | 84 |
| 15 | | Five-Year Outcomes From the Randomized, Phase III Trials CheckMate 017 and 057: Nivolumab Versus Docetaxel in Previously Treated Non-Small-Cell Lung Cancer | Borghaei H | J. Clin. Oncol. | 2021 | 73 |
| 16 | | TCR Repertoire Diversity of Peripheral PD-1(+)CD8(+) T Cells Predicts Clinical Outcomes after Immunotherapy in Patients with Non-Small Cell Lung Cancer | Lu ZM; Wang ZJ; Wang J | Cancer Immunol. Res. | 2020 | 72 |
| 17 | | Multisystem Immune-Related Adverse Events Associated With Immune Checkpoint Inhibitors for Treatment of Non-Small Cell Lung Cancer | Naidoo J | JAMA Oncol. | 2020 | 71 |
| 18 | | Camrelizumab plus carboplatin and pemetrexed versus chemotherapy alone in chemotherapy-naive patients with advanced non-squamous non-small-cell lung cancer (CameL): a randomised, open-label, multicentre, phase 3 trial | Zhou CC | Lancet Resp. Med. | 2021 | 68 |
| 19 | | Changes in CT Radiomic Features Associated with Lymphocyte Distribution Predict Overall Survival and Response to Immunotherapy in Non-Small Cell Lung Cancer | Madabhushi A | Cancer Immunol. Res. | 2020 | 64 |
| 20 | | Adjuvant atezolizumab after adjuvant chemotherapy in resected stage IB-IIIA non-small-cell lung cancer (IMpower010): a randomised, multicentre, open-label, phase 3 trial | Felip E | Lancet | 2021 | 63 |
| 21 | | Updated Overall Survival and PD-L1 Subgroup Analysis of Patients With Extensive-Stage Small-Cell Lung Cancer Treated With Atezolizumab, Carboplatin, and Etoposide (IMpower133) | Liu SV | J. Clin. Oncol. | 2021 | 60 |
| 22 | | Outcomes with durvalumab by tumour PD-L1 expression in unresectable, stage III non-small-cell lung cancer in the PACIFIC trial | Paz-Ares L | Ann. Oncol. | 2020 | 60 |
| 23 | | KEAP1-driven co-mutations in lung adenocarcinoma unresponsive to immunotherapy despite high tumor mutational burden | Maugeri-Sacca M | Ann. Oncol. | 2020 | 55 |
| 24 | | Pembrolizumab in patients with non-small-cell lung cancer of performance status 2 (PePS2): a single arm, phase 2 trial | Middleton G | Lancet Resp. Med. | 2020 | 54 |
| 25 | | Safety and patient-reported outcomes of atezolizumab, carboplatin, and etoposide in extensive-stage small-cell lung cancer (IMpower133): a randomized phase I/III trial | Mansfield AS | Ann. Oncol. | 2020 | 51 |
| 26 | | Patient-reported outcomes following pembrolizumab or placebo plus pemetrexed and platinum in patients with previously untreated, metastatic, non-squamous non-small-cell lung cancer (KEYNOTE-189): a multicentre, double-blind, randomised, placebo-controlled, phase 3 trial | Garassino MC | Lancet Oncol. | 2020 | 50 |
| 27 | | HLA-corrected tumor mutation burden and homologous recombination de fi ciency for the prediction of response to PD -(L)1 blockade in advanced non -small -cell lung cancer patients | Lee SH | Ann. Oncol. | 2020 | 48 |
| 28 | | ARCTIC: durvalumab with or without tremelimumab as third-line or later treatment of metastatic non-small-cell lung cancer | Planchard D | Ann. Oncol. | 2020 | 48 |
| 29 | | Tislelizumab Plus Chemotherapy vs Chemotherapy Alone as First-line Treatment for Advanced Squamous Non-Small-Cell Lung Cancer A Phase 3 Randomized Clinical Trial | Wang J | JAMA Oncol. | 2021 | 46 |
| 30 | | Compartmental Analysis of T-cell Clonal Dynamics as a Function of Pathologic Response to Neoadjuvant PD-1 Blockade in Resectable Non-Small Cell Lung Cancer | Smith KN | Clin. Cancer Res. | 2020 | 44 |
| 31 | | Impact of DNA Damage Response and Repair (DDR) Gene Mutations on Efficacy of PD-(L)1 Immune Checkpoint Inhibition in Non-Small Cell Lung Cancer | Awad MM | Clin. Cancer Res. | 2020 | 43 |
| 32 | | Efficacy and Safety of Pembrolizumab Plus Docetaxel vs Docetaxel Alone in Patients With Previously Treated Advanced Non-Small Cell Lung Cancer The PROLUNG Phase 2 Randomized Clinical Trial | Arrieta O | JAMA Oncol. | 2020 | 42 |
| 33 | | Neoadjuvant durvalumab with or without stereotactic body radiotherapy in patients with early-stage non-small-cell lung cancer: a single-centre, randomised phase 2 trial | Altorki NK | Lancet Oncol. | 2021 | 41 |
| 34 | | CD44 Promotes PD-L1 Expression and Its Tumor-Intrinsic Function in Breast and Lung Cancers | Ursini-Siegel J | Cancer Res. | 2020 | 41 |
| 35 | | Second-line nivolumab in relapsed small-cell lung cancer: CheckMate 331 | Spigel DR | Ann. Oncol. | 2021 | 40 |
| 36 | | Phase 1 Trial of Pembrolizumab Administered Concurrently With Chemoradiotherapy for Locally Advanced Non-Small Cell Lung Cancer A Nonrandomized Controlled Trial | Jabbour SK | JAMA Oncol. | 2020 | 40 |
| 37 | | Clinical activity of programmed cell death 1 (PD-1) blockade in never, light, and heavy smokers with non-small-cell lung cancer and PD-L1 expression >= 50% | Gainor JF | Ann. Oncol. | 2020 | 40 |
| 38 | | Baseline Plasma Tumor Mutation Burden Predicts Response to Pembrolizumab-based Therapy in Patients with Metastatic Non-Small Cell Lung Cancer | Aggarwal C | Clin. Cancer Res. | 2020 | 37 |
| 39 | | Continuous Versus 1-Year Fixed-Duration Nivolumab in Previously Treated Advanced Non-Small-Cell Lung Cancer: CheckMate 153 | Waterhouse DM | J. Clin. Oncol. | 2020 | 36 |
| 40 | | Clarification of Definitions of Hyperprogressive Disease During Immunotherapy for Non-Small Cell Lung Cancer | Caramella C | JAMA Oncol. | 2020 | 35 |
| 41 | | A phase 2 trial of consolidation pembrolizumab following concurrent chemoradiation for patients with unresectable stage III non-small cell lung cancer: Hoosier cancer research network LUN 14-179 | Durm GA | Cancer | 2020 | 34 |
| 42 | | Clinicopathological and genomic correlates of programmed cell death ligand 1 (PD-L1) expression in nonsquamous non-small-cell lung cancer | Awad MM | Ann. Oncol. | 2020 | 34 |
| 43 | | Health-Related Quality of Life With Carboplatin-Paclitaxel or nab-Paclitaxel With or Without Pembrolizumab in Patients With Metastatic Squamous Non-Small-Cell Lung Cancer | Mazieres J | J. Clin. Oncol. | 2020 | 32 |
| 44 | | Pembrolizumab Plus Ipilimumab or Placebo for Metastatic Non-Small-Cell Lung Cancer With PD-L1 Tumor Proportion Score >= 50%: Randomized, Double-Blind Phase III KEYNOTE-598 Study | Boyer M | J. Clin. Oncol. | 2021 | 31 |
| 45 | | Association of Probiotic Clostridium butyricum Therapy with Survival and Response to Immune Checkpoint Blockade in Patients with Lung Cancer | Tomita Y | Cancer Immunol. Res. | 2020 | 31 |
| 46 | | Pembrolizumab plus allogeneic NK cells in advanced non-small cell lung cancer patients | Jiang Y | J. Clin. Invest. | 2020 | 30 |
| 47 | | Real-world use and survival outcomes of immune checkpoint inhibitors in older adults with non-small cell lung cancer | Youn B | Cancer | 2020 | 30 |
| 48 | | Nivolumab and Ipilimumab as Maintenance Therapy in Extensive-Disease Small-Cell Lung Cancer: CheckMate 451 | Owonikoko TK | J. Clin. Oncol. | 2021 | 29 |
| 49 | | Circulating T-cell Immunosenescence in Patients with Advanced Non-small Cell Lung Cancer Treated with Single-agent PD-1/PD-L1 Inhibitors or Platinum-based Chemotherapy | Chaput N | Clin. Cancer Res. | 2021 | 28 |
| 50 | | SAKK 16/14: Durvalumab in Addition to Neoadjuvant Chemotherapy in Patients With Stage IIIA(N2) Non-Small-Cell Lung Cancer-A Multicenter Single-Arm Phase II Trial | Rothschild SI | J. Clin. Oncol. | 2021 | 27 |
| 51 | | Biomarkers Associated with Beneficial PD-1 Checkpoint Blockade in Non-Small Cell Lung Cancer (NSCLC) Identified Using High-Plex Digital Spatial Profiling | Zugazagoitia J | Clin. Cancer Res. | 2020 | 27 |
| 52 | | Tumor-infiltrating lymphocyte treatment for anti-PD-1-resistant metastatic lung cancer: a phase 1 trial | Creelan BC | Nat. Med. | 2021 | 22 |
| 53 | | Safety and Patient-Reported Outcomes of Atezolizumab Plus Chemotherapy With or Without Bevacizumab Versus Bevacizumab Plus Chemotherapy in Non-Small-Cell Lung Cancer | Reck M | J. Clin. Oncol. | 2020 | 22 |
| 54 | | Performance status and end-of-life care among adults with non-small cell lung cancer receiving immune checkpoint inhibitors | Gainor JF | Cancer | 2020 | 22 |
| 55 | | A Phase 1 study of gefitinib combined with durvalumab in EGFR TKI-naive patients withEGFRmutation-positive locally advanced/metastatic non-small-cell lung cancer | Creelan BC | Br. J. Cancer | 2021 | 21 |
| 56 | | Pembrolizumab Plus Concurrent Chemoradiation Therapy in Patients With Unresectable, Locally Advanced, Stage III Non-Small Cell Lung Cancer The Phase 2 KEYNOTE-799 Nonrandomized Trial | Jabbour SK | JAMA Oncol. | 2021 | 20 |
| 57 | | SHP-2 and PD-L1 Inhibition Combined with Radiotherapy Enhances Systemic Antitumor Effects in an Anti-PD-1-Resistant Model of Non-Small Cell Lung Cancer | Welsh JW | Cancer Immunol. Res. | 2020 | 19 |
| 58 | | Generation of Genetically Engineered Mouse Lung Organoid Models for Squamous Cell Lung Cancers Allows for the Study of Combinatorial Immunotherapy | Hai J | Clin. Cancer Res. | 2020 | 18 |
| 59 | | Nivolumab with carboplatin, paclitaxel, and bevacizumab for first-line treatment of advanced nonsquamous non-small-cell lung cancer | Nakagawa K | Ann. Oncol. | 2021 | 16 |
| 60 | | Amplifying Outcomes: Checkpoint Inhibitor Combinations in First-Line Non-Small Cell Lung Cancer | Melosky B | Oncologist | 2020 | 16 |
| 61 | | Lipopolysaccharide-Mediated Chronic Inflammation Promotes Tobacco Carcinogen-Induced Lung Cancer and Determines the Efficacy of Immunotherapy | Di YP | Cancer Res. | 2021 | 15 |
| 62 | | Phase Ib Study of Crizotinib Plus Pembrolizumab in Patients with Previously Untreated Advanced Non-Small Cell Lung Cancer with ALK Translocation | Patel SP | Oncologist | 2020 | 15 |
| 63 | | Association of the prognostic model iSEND with PD-1/L1 monotherapy outcome in non-small-cell lung cancer | Lopes G | Br. J. Cancer | 2020 | 14 |
| 64 | | Five-Year Survival Outcomes From the PACIFIC Trial: Durvalumab After Chemoradiotherapy in Stage III Non-Small-Cell Lung Cancer | Spigel DR | J. Clin. Oncol. | 2022 | 12 |
| 65 | | Resistance to Durvalumab and Durvalumab plus Tremelimumab Is Associated with Functional STK11 Mutations in Patients with Non-Small Cell Lung Cancer and Is Reversed by STAT3 Knockdown | Ascierto ML; Oberst MD | Cancer Discov. | 2021 | 12 |
| 66 | | FDA Approval Summary: Atezolizumab and Durvalumab in Combination with Platinum-Based Chemotherapy in Extensive Stage Small Cell Lung Cancer | Mathieu L | Oncologist | 2021 | 12 |
| 67 | | Safety and efficacy of quavonlimab, a novel anti-CTLA-4 antibody (MK-1308), in combination with pembrolizumab in first-line advanced non-small-cell lung cancer | Cho BC | Ann. Oncol. | 2021 | 12 |
| 68 | | CCL7 recruits cDC1 to promote antitumor immunity and facilitate checkpoint immunotherapy to non-small cell lung cancer | Zhong B | Nat. Commun. | 2020 | 12 |
| 69 | | Immunotherapy-Mediated Thyroid Dysfunction: Genetic Risk and Impact on Outcomes with PD-1 Blockade in Non-Small Cell Lung Cancer | Hellmann MD | Clin. Cancer Res. | 2021 | 11 |
| 70 | | Th17 cells contribute to combination MEK inhibitor and anti-PD-L1 therapy resistance in KRAS/p53 mutant lung cancers | Gibbons DL | Nat. Commun. | 2021 | 11 |
| 71 | | Intestinal Akkermansia muciniphila predicts clinical response to PD-1 blockade in patients with advanced non-small-cell lung cancer | Zitvogel L | Nat. Med. | 2022 | 10 |
| 72 | | Notch signaling and efficacy of PD-1/PD-L1 blockade in relapsed small cell lung cancer | Thomas A | Nat. Commun. | 2021 | 10 |
| 73 | | Clinical activity of a htert (vx-001) cancer vaccine as post-chemotherapy maintenance immunotherapy in patients with stage IV non-small cell lung cancer: final results of a randomised phase 2 clinical trial | Gridelli C | Br. J. Cancer | 2020 | 9 |
| 74 | | Neoadjuvant Nivolumab plus Chemotherapy in Resectable Lung Cancer | Forde PM | N. Engl. J. Med. |  | 8 |
| 75 | | Antibiotic-exposed patients with non-small-cell lung cancer preserve efficacy outcomes following first-line chemo-immunotherapy | Cortellini A | Ann. Oncol. | 2021 | 8 |
| 76 | | A Phase I Study of APX005M and Cabiralizumab with or without Nivolumab in Patients with Melanoma, Kidney Cancer, or Non-Small Cell Lung Cancer Resistant to Anti-PD-1/PD-L1 | Weiss SA | Clin. Cancer Res. | 2021 | 8 |
| 77 | | Cooperative Targeting of Immunotherapy-Resistant Melanoma and Lung Cancer by an AXL-Targeting Antibody-Drug Conjugate and Immune Checkpoint Blockade | Peeper DS | Cancer Res. | 2021 | 8 |
| 78 | | Prolonging Survival: The Role of Immune Checkpoint Inhibitors in the Treatment ofExtensive-StageSmall Cell Lung Cancer | Melosky B | Oncologist | 2020 | 8 |
| 79 | | First-in-human phase 1 study of the anti-TIGIT antibody vibostolimab as monotherapy or with pembrolizumab for advanced solid tumors, including non-small-cell lung cancer | Niu J | Ann. Oncol. | 2022 | 7 |
| 80 | | Consolidation nivolumab and ipilimumab versus observation in limited-disease small-cell lung cancer after chemo-radiotherapy - results from the randomised phase II ETOP/IFCT 4-12 STIMULI trial | Stahel RA | Ann. Oncol. | 2022 | 6 |
| 81 | | Nivolumab Plus Ipilimumab vs Nivolumab for Previously Treated Patients With Stage IV Squamous Cell Lung Cancer The Lung-MAP S1400I Phase 3 Randomized Clinical Trial | Gettinger SN | JAMA Oncol. | 2021 | 6 |
| 82 | | Nivolumab Plus Ipilimumab vs Nivolumab for Previously Treated Patients With Stage IV Squamous Cell Lung Cancer The Lung-MAP S14001 Phase 3 Randomized Clinical Trial | Gettinger SN | JAMA Oncol. |  | 6 |
| 83 | | KEYNOTE-032: A Randomized Phase I Study of Pembrolizumab in Chinese Patients with Advanced Non-Small Cell Lung Cancer | Zhang L | Oncologist | 2020 | 6 |
| 84 | | Efficacy of first-line atezolizumab combination therapy in patients with non-small cell lung cancer receiving proton pump inhibitors: post hoc analysis of IMpower150 | Hopkins AM | Br. J. Cancer | 2022 | 5 |
| 85 | | Nodal immune flare mimics nodal disease progression following neoadjuvant immune checkpoint inhibitors in non-small cell lung cancer | Cascone T; Heymach JV | Nat. Commun. | 2021 | 5 |
| 86 | | A Serum Protein Classifier Identifying Patients with Advanced Non-Small Cell Lung Cancer Who Derive Clinical Benefit from Treatment with Immune Checkpoint Inhibitors | Smit EF | Clin. Cancer Res. | 2020 | 5 |
| 87 | | Sugemalimab versus placebo, in combination with platinum-based chemotherapy, as first-line treatment of metastatic non-small-cell lung cancer (GEMSTONE-302): interim and final analyses of a double-blind, randomised, phase 3 clinical trial | Zhou CC | Lancet Oncol. | 2022 | 4 |
| 88 | | Clinical definition of acquired resistance to immunotherapy in patients with metastatic non-small-cell lung cancer | Hellmann MD | Ann. Oncol. | 2021 | 4 |
| 89 | | Dysregulation of T-FH-B-T-RM lymphocyte cooperation is associated with unfavorable anti-PD-1 responses in EGFR-mutant lung cancer | Lee I | Nat. Commun. | 2021 | 4 |
| 90 | | JASPER: Phase 2 trial of first-line niraparib plus pembrolizumab in patients with advanced non-small cell lung cancer | Ramalingam SS | Cancer |  | 4 |
| 91 | | Efficacy and safety of immune checkpoint blockade in self-identified black patients with advanced non-small cell lung cancer | Owonikoko TK | Cancer | 2020 | 4 |
| 92 | | Blood-based tumor mutational burden as a biomarker for atezolizumab in non-small cell lung cancer: the phase 2 B-F1RST trial | Velcheti V | Nat. Med. | 2022 | 3 |
| 93 | | Intratumoral plasma cells predict outcomes to PD-L1 blockade in non-small cell lung cancer | Patil NS; Nabet BY | Cancer Cell | 2022 | 3 |
| 94 | | Durvalumab plus tremelimumab alone or in combination with low-dose or hypofractionated radiotherapy in metastatic non-small-cell lung cancer refractory to previous PD(L)-1 therapy: an open-label, multicentre, randomised, phase 2 trial | Schoenfeld JD | Lancet Oncol. | 2022 | 3 |
| 95 | | Sugemalimab versus placebo after concurrent or sequential chemoradiotherapy in patients with locally advanced, unresectable, stage III non-small-cell lung cancer in China (GEMSTONE-301): interim results of a randomised, double-blind, multicentre, phase 3 trial | Wu YL | Lancet Oncol. | 2022 | 3 |
| 96 | | Platelet PD-L1 reflects collective intratumoral PD-L1 expression and predicts immunotherapy response in non-small cell lung cancer | Zender L | Nat. Commun. | 2021 | 3 |
| 97 | | Neoadjuvant Gene-Mediated Cytotoxic Immunotherapy for Non-Small-Cell Lung Cancer: Safety and Immunologic Activity | Albelda SM | Mol. Ther. | 2021 | 3 |
| 98 | | Impact of preexisting antinuclear antibodies on combined immunotherapy and chemotherapy in advanced non-small cell lung cancer patients | Yamada T | Med. Oncol. | 2020 | 3 |
| 99 | | Lack of Association Between Radiographic Tumor Burden and Efficacy of Immune Checkpoint Inhibitors in Advanced Lung Cancer | Gerber DE | Oncologist | 2020 | 3 |
| 100 | | Association of PD-1/PD-L1 Co-location with Immunotherapy Outcomes in Non-Small Cell Lung Cancer | Rimm DL | Clin. Cancer Res. | 2022 | 2 |
| 101 | | A Phase Ib/II Study of Pepinemab in Combination with Avelumab in Advanced Non-Small Cell Lung Cancer | Fisher TL | Clin. Cancer Res. | 2021 | 2 |
| 102 | | First-Line Durvalumab in Addition to Etoposide and Platinum for Extensive-Stage Small Cell Lung Cancer: A US-Based Cost-Effectiveness Analysis | Xu XW; Weng XH | Oncologist | 2021 | 1 |
| 103 | | A Phase Ib Open-Label, Multicenter Study of Inhaled DV281, a TLR9 Agonist, in Combination with Nivolumab in Patients with Advanced or Metastatic Non-small Cell Lung Cancer | Garon EB | Clin. Cancer Res. | 2021 | 1 |
| 104 | | Immunotherapy for Metastatic Non-Small Cell Lung Cancer: Real-World Data from an Academic Central and Eastern European Center | Ivanovic M | Oncologist |  | 1 |
| 105 | | 11 beta hydroxysteroid dehydrogenase 1: a new marker for predicting response to immune-checkpoint blockade therapy in non-small-cell lung carcinoma | Saito R | Br. J. Cancer | 2020 | 1 |
| 106 | | Association of CD274 (PD-L1) Copy Number Changes with Immune Checkpoint Inhibitor Clinical Benefit in Non-Squamous Non-Small Cell Lung Cancer | Huang RSP | Oncologist |  | 0 |
| 107 | | Targeting brain lesions of non-small cell lung cancer by enhancing CCL2-mediated CAR-T cell migration | Dotti G; Du HW | Nat. Commun. | 2022 | 0 |
| 108 | | Multicenter Real-World Study on Effectiveness and Early Discontinuation Predictors in Patients With Non-small Cell Lung Cancer Receiving Nivolumab | Pasello G | Oncologist | 2022 | 0 |
| 109 | | Long-term outcomes in patients with advanced and/or metastatic non-small cell lung cancer who completed 2 years of immune checkpoint inhibitors or achieved a durable response after discontinuation without disease progression: Multicenter, real-world data (KCSG LU20-11) | Ahn MJ | Cancer | 2022 | 0 |
| 110 | | Safety and Efficacy of First-Line Pembrolizumab in Black Patients with Metastatic Non-Small Cell Lung Cancer | Peravali M | Oncologist | 2021 | 0 |
