# Supplementary material for: Global research landscape and trends of lung cancer immunotherapy: A bibliometric analysis
Source: Front Immunol. 2022 Dec 1;13:1032747. doi: 10.3389/fimmu.2022.1032747 (PMC9751816; doi:10.3389/fimmu.2022.1032747)
Supplement: Supplementary file 9 [file Table_5.docx]

| **TABLE S5** \| The corresponding authors with at least two top-papers in lung cancer immunotherapy from 2010 to 2022. | | | | | |
| --- | --- | --- | --- | --- | --- |
| **Corresponding author** | **Paper number** | **Total citation** | **Average citations per paper** | **Top-paper number** | **Representative work^a^** |
| Hellmann MD | 14 | 5394 | 385.29 | 7 | Nivolumab plus Ipilimumab in Lung Cancer with a High Tumor Mutational Burden |
| Rizvi NA | 5 | 2103 | 420.60 | 4 | Activity and safety of nivolumab, an anti-PD-1 immune checkpoint inhibitor, for patients with advanced, refractory squamous non-small-cell lung cancer (CheckMate 063): a phase 2, single-arm trial |
| Reck M | 12 | 2093 | 174.42 | 4 | Updated Analysis of KEYNOTE-024: Pembrolizumab Versus Platinum-Based Chemotherapy for Advanced Non-Small-Cell Lung Cancer With PD-L1 Tumor Proportion Score of 50% or Greater |
| Paz-Ares L | 6 | 2483 | 413.83 | 3 | Pembrolizumab plus Chemotherapy for Squamous Non-Small-Cell Lung Cancer |
| Gettinger S | 3 | 988 | 329.33 | 3 | Five-Year Follow-Up of Nivolumab in Previously Treated Advanced Non-Small-Cell Lung Cancer: Results From the CA209-003 Study |
| Brahmer JR | 4 | 9966 | 2491.50 | 2 | Pembrolizumab versus Chemotherapy for PD-L1-Positive Non-Small-Cell Lung Cancer |
| Garon EB | 8 | 4280 | 535.00 | 2 | Pembrolizumab for the Treatment of Non-Small-Cell Lung Cancer |
| Horn L | 4 | 1637 | 409.25 | 2 | First-Line Atezolizumab plus Chemotherapy in Extensive-Stage Small-Cell Lung Cancer |
| Rimm DL | 5 | 1179 | 235.80 | 2 | Quantitative Assessment of the Heterogeneity of PD-L1 Expression in Non-Small-Cell Lung Cancer |
| Cappuzzo F | 4 | 1033 | 258.25 | 2 | Atezolizumab in combination with carboplatin plus nab-paclitaxel chemotherapy compared with chemotherapy alone as first-line treatment for metastatic non-squamous non-small-cell lung cancer (IMpower130): a multicentre, randomised, open-label, phase 3 trial |
| Okamoto I | 5 | 830 | 166.00 | 2 | Association of PD-L1 overexpression with activating EGFR mutations in surgically resected nonsmall- cell lung cancer |
| Hayashi H | 5 | 796 | 159.20 | 2 | Association of Immune-Related Adverse Events With Nivolumab Efficacy in Non-Small Cell Lung Cancer |
| Besse B | 6 | 779 | 129.83 | 2 | Hyperprogressive Disease in Patients With Advanced Non-Small Cell Lung Cancer Treated With PD-1/PD-L1 Inhibitors or With Single-Agent Chemotherapy |
| Wu YL | 12 | 793 | 66.08 | 2 | Potential Predictive Value of TP53 and KRAS Mutation Status for Response to PD-1 Blockade Immunotherapy in Lung Adenocarcinoma |
| ^a^The most cited paper in lung cancer immunotherapy of the corresponding author. | | | | | |
